# Supplementary material for: Navigating the river(s) of systems change: a multi-methods, qualitative evaluation exploring the implementation of a systems approach to physical activity in Gloucestershire, England
Source: BMJ Open. 2022 Aug 8;12(8):e063638. doi: 10.1136/bmjopen-2022-063638 (PMC9364398; doi:10.1136/bmjopen-2022-063638)
Supplement: Supplementary data [file bmjopen-2022-063638supp001.pdf]

Supplement I - COREQ Checklist: Nobles et al. Implementing a Whole Systems Approach to Increase Physical Activity

Domain 1: Research team and reflexivity

Personal Characteristics

1. *Interviewer/facilitator*

James Nobles

2. *Credentials*

Ph.D.

3. *Occupation*

Research Fellow in Behavioural and Qualitative Science at the University of Bristol.

4. *Gender*

Male.

5. *Experience and training*

Seven years of qualitative research experience.

Relationship with participants

6. *Relationship established*

A relationship was present with some of the people who were interviewed / worked with for this project. James Nobles was an embedded researcher at Active Gloucestershire (the organisation who facilitate WCM), and so spent one day per week initially in their offices. During this time, he attended many meetings, workshops and events, at which he met some of the people who were interviewed. In all instances, the (prospective) interviewee knew that James' role was that of an evaluator and that he did not work for Active Gloucestershire. A concise explanation of this is offered on page 5, section 2.1.

7. *Participant knowledge of the Interviewer*

Where relationships were already established prior to the interview taking place, the researcher had always made clear that they were evaluating the implementation of WCM. James spoke about the different aspects of the evaluation (e.g. the research questions, the methods, the outputs etc..) at these meetings. It was important that stakeholders, some of which were future interviewees, understood why James was present in the meetings, workshops or events. A concise explanation of this is offered on page 5, section 2.1.

8. *Interviewer characteristics*

James had a pre-existing interest in the design, delivery and evaluation of systems approaches in Public Health. He had previously been involved in the creation of a toolkit that Local Government Authorities in England could use to design their own whole systems approach to prevent and

manage overweight and obesity. The topic of physical activity was somewhat new prior to starting the evaluation of WCM in April 2019, however other members of the team are international experts in physical activity research. Moreover, he – and the wider project team – were aware that the previous approaches to improving physical activity had largely been ineffective in bringing about long-term, sustainable behaviour change. It is worth stating here though that the purpose of the WCM evaluation was not to determine if the population levels of physical activity changed as a result of its delivery, but rather it was mainly concerned with the learning that arises from the implementation of WCM.

Domain 2: Study design

Theoretical framework

9. *Methodological orientation and Theory* - What methodological orientation was stated to underpin the study? e.g. grounded theory, discourse analysis, ethnography, phenomenology, content analysis

This is a multi-method qualitative evaluation study analysed using a framework and content approach. A concise explanation of this is offered on page 5, section 2.1.

Participant selection

10. *Sampling*

All interview participants were purposefully sampled, via one of three means:

- First, the researcher worked with members of the Active Gloucestershire team to identify people who had been involved in, or affected by, the WCM programme.
- Second, the researcher analysed the Ripple Effects Mapping outputs to identify other people or organisations who may have been involved in the periphery of WCM. Ripple Effects Mapping has been described in the main paper.
- Third, the researcher conducted a secondary analysis of a stakeholder survey that was disseminated by Active Gloucestershire amongst their contacts. The researcher looked at the responses to open-ended questions. These respondents were then sent an invitation to partake in an interview by a member of the Active Gloucestershire team.

Ripple Effects Mapping participants comprised of implementation staff (i.e. those involved in the design and delivery of We Can Move). A small group of community residents also took part in an REM workshop, as described in the paper, and all were involved in the implementation of the community-based initiative.

A concise explanation of this is offered on page 7-8, section 2.3.

11. *Method of approach*

All participants were contacted via email in the first instance. They were provided with an overview of the evaluation, a description around the purpose of the interview, and then further information was included in a participant information sheet. Those who were then interested in the study were sent a consent form to complete. If participants had further questions, they were able to contact the researcher directly via phone or video-call software.

The ethical approval statement is provided on page 26.

12. *Sample size*

Interview data from thirty- one people were included in this study.

The justification for this sample size is provided on page 7, section 2.3.

13. *Non-participation* - How many people refused to participate or dropped out? Reasons?

No one refused to take part in an interview or REM session as far as we recall.

Setting

14. *Setting of data collection*

All interview data was collected either over the phone (i.e. telephone interviews) or via video-call software (e.g. Microsoft Teams or Zoom).

Initial REM sessions were completed via in-person workshops, and follow up workshops were all completed online using Microsoft Teams or Zoom.

A concise explanation of this is offered on page 7, section 2.3.

15. *Presence of non-participants* - Was anyone else present besides the participants and researchers?

No

16. *Description of sample* - What are the important characteristics of the sample? e.g. demographic data, date

The reporting of this is offered on page 10, section 3.

Data collection

17. *Interview guide* - Were questions, prompts, guides provided by the authors? Was it pilot tested?

The interview guide is provided as a supplement to the manuscript. The interview guide was developed by the co-authors of the paper who have extensive experience in qualitative and evaluation research. The questions were designed so as to specifically probe into areas of interest. The REM outputs, as described in the paper, were also used to probe for further information in the interviews.

A concise statement of this is provided on page 7, section 2.3.

18. *Repeat interviews* - Were repeat interviews carried out? If yes, how many?

No

19. *Audio/visual recording* - Did the research use audio or visual recording to collect the data?

Yes – interviews were audio recorded, and REM workshops (online only) were visually and audio recorded.

Concise statements of this are provided on page 7-8, section 2.3.

20. *Field notes* - Were field notes made during and/or after the interview or focus group?

Yes. These were predominantly made to facilitate or bolster the lines of inquiry when carrying out the interview (e.g. notes made on topics discussed so that aspects of the conversation could be revisited in the interview if necessary). Notes were not used to aid the analysis.

21. *Duration* - What was the duration of the interviews or focus

group? A concise statement of this is provided on page 7, section 2.3.

22. *Data saturation* - Was data saturation discussed?

No, we planned to complete a pragmatic number of interviews per case study (n=10).

23. *Transcripts returned* - Were transcripts returned to participants for comment and/or correction?

No, although upon receiving the transcripts, authors read these concurrent to the audio recording to ensure their accuracy.

Domain 3: analysis and findings

Data analysis

24. *Number of data coders* - How many data coders coded the data?

One primary coder and a second (CF) completed a separate analysis of the data from one case study. The two authors discussed their findings and came to an agreement on the most appropriate way to present these findings.

A concise statement of this is provided on page 9, section 2.4.

25. *Description of the coding tree* - Did authors provide a description of the coding tree?

No

26. *Derivation of themes* - Were themes identified in advance or derived from the data?

Yes – some themes were created based upon the main areas of exploration (e.g. mechanisms of change, contextual factors, impacts etc…), and others were derived from the data where required.

A concise explanation of this is provided on page 9, section 2.4.

27. *Software* - What software, if applicable, was used to manage the data?

~~NVIVO12~~NVivo12

28. *Participant checking* - Did participants provide feedback on the findings?

We reported the findings of this work back to the implementation team and wider stakeholders to ensure that it resonated with what they had experienced. Further details are available in the manuscript.

A concise statement of this is provided on page 9, section 2.4.

Reporting

29. *Quotations presented* - Were participant quotations presented to illustrate the themes / findings? Was each quotation identified? e.g. participant number

30. *Data and findings consistent* - Was there consistency between the data presented and the findings?

31. *Clarity of major themes* - Were major themes clearly presented in the findings?

N/A – this is based upon reviewer comments.

32. *Clarity of minor themes* - Is there a description of diverse cases or discussion of minor themes N/A – this is based upon reviewer comments.
