# Supplementary material for: Navigating the river(s) of systems change: a multi-methods, qualitative evaluation exploring the implementation of a systems approach to physical activity in Gloucestershire, England
Source: BMJ Open. 2022 Aug 8;12(8):e063638. doi: 10.1136/bmjopen-2022-063638 (PMC9364398; doi:10.1136/bmjopen-2022-063638)
Supplement: Supplementary data [file bmjopen-2022-063638supp002.pdf]

Evaluation of We Can Move

Supplement II: Interview Topic Guide

1. Can you tell me about your **role** or involvement in "we can move"?

- How did your involvement commence?
- What were your reasons for getting involved?
- If not knowledgeable of "we can move", the role with Active Gloucestershire

2. What do you **perceive "we can move" to be**?

- What do you think of when you hear "we can move"?
- What do you know about it?

3. What were your **expectations** from "we can move"?

- How did you expect it to unfold?

4. How have you seen "we can move" **evolve over time**?

- How has this happened?
- What has contributed to these adaptations to occur?

5. What have been the **main successes** of "we can move" from your perspective?

- Why did you highlight these examples?

6. To what extent do you feel that "we can move" is **focused on the right things**?

7. [Facilitators] What has **helped** "we can move" to work as it does?

- i.e. what has helped to make it successful?
- What are the important ingredients?

[Barriers] What are the **challenges**, or have been the challenges, facing "we can move"?

- What has made it more difficult?

Contextual factors that may influence the projects?

8. What **impacts have you experienced** or heard about regarding "we can move"?

- i.e. impacts on self, society, organisation, policy etc….
- Expected or unexpected?
- How did the impact occur?
- Are these as you expected?
- Did they lead to any additional impacts?

9. For you, how could "we can move" **improve** or develop in the **future**?

- What would be required to help achieve this?

10. What do you **perceive your role to be** in "we can move" in the future?
