# Supplementary material for: Navigating the river(s) of systems change: a multi-methods, qualitative evaluation exploring the implementation of a systems approach to physical activity in Gloucestershire, England
Source: BMJ Open. 2022 Aug 8;12(8):e063638. doi: 10.1136/bmjopen-2022-063638 (PMC9364398; doi:10.1136/bmjopen-2022-063638)
Supplement: Author's manuscript [file bmjopen-2022-063638.draft_revisions.pdf]

BMJ Open is committed to open peer review. As part of this commitment we make the peer review history of every article we publish publicly available.

When an article is published we post the peer reviewers' comments and the authors' responses online. We also post the versions of the paper that were used during peer review. These are the versions that the peer review comments apply to.

The versions of the paper that follow are the versions that were submitted during the peer review process. They are not the versions of record or the final published versions. They should not be cited or distributed as the published version of this manuscript.

BMJ Open is an open access journal and the full, final, typeset and author-corrected version of record of the manuscript is available on our site with no access controls, subscription charges or pay-per-view fees (http://bmjopen.bmj.com).

If you have any questions on BMJ Open's open peer review process please email info.bmjopen@bmj.com

BMJ Open

Navigating the river(s) of systems change: a multi-methods, qualitative evaluation exploring the implementation of a systems approach to physical activity in Gloucestershire, England

Journal:	*BMJ Open*
Manuscript ID	bmjopen-2022-063638
Article Type:	Original research
Date Submitted by the Author:	13-Apr-2022

Complete List of Authors:	Nobles, James; University of Bristol, Population Health Sciences; NIHR ARC West<br>Fox, Charlotte; Royal Borough of Windsor and Maidenhead Council<br>Inman-Ward, Alan; Active Gloucestershire<br>Beasley, Tom; Active Gloucestershire<br>Redwood, Sabi; Bristol, Population Health Sciences; NIHR ARC West<br>Jago, Russ; University of Bristol, Centre for Exercise, Nutrition and Health Sciences; NIHR ARC West<br>Foster, Charlie ; University of Bristol Faculty of Health Sciences

Keywords:	PUBLIC HEALTH, Health policy < HEALTH SERVICES ADMINISTRATION & MANAGEMENT, Organisation of health services < HEALTH SERVICES ADMINISTRATION & MANAGEMENT, PREVENTIVE MEDICINE, QUALITATIVE RESEARCH, SOCIAL MEDICINE

SCHOLARONE™
Manuscripts

I, the Submitting Author has the right to grant and does grant on behalf of all authors of the Work (as defined in the below author licence), an exclusive licence and/or a non-exclusive licence for contributions from authors who are: i) UK Crown employees; ii) where BMJ has agreed a CC-BY licence shall apply, and/or iii) in accordance with the terms applicable for US Federal Government officers or employees acting as part of their official duties; on a worldwide, perpetual, irrevocable, royalty-free basis to BMJ Publishing Group Ltd ("BMJ") its licensees and where the relevant Journal is co-owned by BMJ to the co-owners of the Journal, to publish the Work in this journal and any other BMJ products and to exploit all rights, as set out in our licence.

The Submitting Author accepts and understands that any supply made under these terms is made by BMJ to the Submitting Author unless you are acting as an employee on behalf of your employer or a postgraduate student of an affiliated institution which is paying any applicable article publishing charge ("APC") for Open Access articles. Where the Submitting Author wishes to make the Work available on an Open Access basis (and intends to pay the relevant APC), the terms of reuse of such Open Access shall be governed by a Creative Commons licence – details of these licences and which Creative Commons licence will apply to this Work are set out in our licence referred to above.

Other than as permitted in any relevant BMJ Author's Self Archiving Policies, I confirm this Work has not been accepted for publication elsewhere, is not being considered for publication elsewhere and does not duplicate material already published. I confirm all authors consent to publication of this Work and authorise the granting of this licence.

For peer review only

Title

Navigating the river(s) of systems change: a multi-methods, qualitative evaluation exploring the implementation of a systems approach to physical activity in Gloucestershire, England

Authors and institutions

James Nobles[1,2 *], Charlotte Fox[3], Alan Inman-Ward[4], Tom Beasley[4], Sabi Redwood[1,2], Russell Jago[1,2,5], and Charlie Foster[5]

[1] The National Institute for Health Research Applied Research Collaboration West (NIHR ARC West) at University Hospitals Bristol National Health Service Foundation Trust, United Kingdom

[2] Population Health Sciences, Bristol Medical School, University of Bristol, United Kingdom

[3] Royal Borough of Windsor and Maidenhead Council, United Kingdom

[4] Active Gloucestershire, Gloucester, United Kingdom

[5] Centre for Exercise, Nutrition and Health Sciences, School for Policy Studies, University of Bristol, United Kingdom

*Corresponding author: James Nobles, University of Bristol, james.nobles@bristol.ac.uk

Structured abstract

Objectives: Systems approaches aim to change the environments in which people live, through cross-sectoral working, by harnessing the complexity of the problem. This paper sought to identify: a) the mechanisms of implementation in We Can Move (WCM), b) the barriers to implementation, c) key contextual factors that influence implementation, and d) impacts associated with WCM.

Design: A multi-methods evaluation of WCM was completed between April 2019 and April 2021. Ripple Effects Mapping (REM) and semi-structured interviewers were used. Framework and content analysis were systematically applied to the dataset.

Setting: We Can Move - a systems approach being implemented in Gloucestershire, England.

Participants: 31 stakeholder interviews and 25 stakeholders involved in 15 REM workshops.

Results: A white-water rafting analogy was developed to present the main findings. The successful implementation of WCM required a facilitative, well-connected, and knowledgeable guide (i.e. the

For peer review only - http://bmjopen.bmj.com/site/about/guidelines.xhtml

lead organisation), a crew (i.e. wider stakeholders) who's vision and agenda aligned with WCM's purpose, and a flexible delivery approach that could respond to ever-changing nature of the river (i.e. local and national circumstances). The context surrounding WCM further strengthened and hampered its implementation. Barriers included evaluative difficulties, a difference in stakeholder and organisational perspectives, misaligned expectations and understandings of WCM, and COVID-19 implications. WCM was said to strengthen cohesion and collaboration between partners, benefit other agendas and policies (e.g. mental health, town planning, inequality), and improve physical activity opportunities and environments.

Conclusions: This paper is one of the first to evaluate a systems approach to increasing physical activity. We highlight key mechanistic and contextual factors that influenced the implementation of WCM and demonstrate some of the wider benefits from such approaches. Further research and methodologies are required to build the evidence base surrounding systems approaches in public health.

Key words

Systems approach; systems science; complex adaptive systems; public health; participatory research; ripple effects mapping; multi-method

Strengths and limitations of this study

- A multi-methods, innovative and realist-informed approach was used to comprehensively answer the evaluative aims of this research.
- Ripple Effects Mapping was used to evidence wider impacts of WCM, but also to inform the interviewee sampling and questioning.
- The data collection approach was predominantly completed virtually due to COVID-19 restrictions which lessened the utility of REM.

Word count: 7809

For peer review only

Main text

1. Introduction

Global data suggest that a quarter of adults and three quarters of adolescents are not engaged in recommended amounts of physical activity (1, 2). National data in England paint a similar picture. Physical activity behaviour is socially patterned, and people who live in more deprived areas and who are from ethnically diverse groups have lower levels of physical activity (3). These low physical activity levels can contribute towards poor health and wellbeing outcomes (4-6), as well as having notable direct and indirect health and social costs (4, 7).

The World Health Organisation recently explored the complex drivers of low physical activity (8). They argued that the population levels of physical activity are the outcome of an interconnected, interdependent, and evolving web of causal factors – in other words, a complex adaptive system. They outlined 45 causal factors that spanned multiple themes; from individual factors (e.g. motivation, self-efficacy, family support), to socio-political factors (e.g. employment status, socio-economic status, disposable income) to transport and environmental factors (e.g. public transport, air quality, walking infrastructure) (8). Others, including a 2021 commission by the Lancet, corroborate these arguments (6, 9-13).

Historically, interventions to increase physical activity have largely focused on individual agency and decision-making as the primary mechanism of change (24). These interventions seek to change individual behaviours, either through short-term educational approaches or through the provision of physical activity opportunities (14). These approaches are also politically appealing because they require minimal political will to implement, they are relatively low cost, they are easy to measure, and there is an evidence base that can be used to justify their delivery (15-20). However, individual level interventions can exacerbate, rather than reduce, health inequalities, and seldom change the environments that people move and function within (15, 17, 21).

Approaches that require lower levels of individual agency have the potential for broader reach, and more importantly, reducing inequalities (15). A systems approach aims to influence multiple parts of the system concurrently by drawing on the expertise and resources of individuals, communities, organisations, and sectors (6, 22, 23). Systems approaches have the potential to reshape the environments that people interact with, and in doing so, can change default and automatic behaviours (11, 15, 22, 24, 25). These approaches can adapt over time in response to dynamic contexts, the engagement from stakeholders, and the observed impacts (intended or unintended) across the system (6). Therefore, no two systems approaches will look, function, or feel the same. There is broad

For peer review only

For peer review only - http://bmjopen.bmj.com/site/about/guidelines.xhtml

consensus that systems approaches are required to help address many of the complex public health issues being faced (8, 11, 18, 23, 26-29).

Practice has now outstripped the research and evidence base. During the last 10 years, there has been a groundswell in the implementation of systems approaches (27, 30). For example, Sport England – a national non-departmental government organisation – has allocated over £100 million for Local Delivery Pilots. The purpose of the Local Delivery Pilots are to test out novel, systemic, and joined up approaches that have the long-term ambition of improving population physical activity, and health, outcomes (29). The Office for Health Improvement and Disparities (formerly Public Health England) has also invested substantial resource to support local government authorities to implement a whole systems approach to obesity (26, 31). Similar patterns and investments have been observed internationally (27, 30). Despite the growing interest in, and implementation of, systems approaches in Public Health, there is a distinct lack of published research surrounding their evaluation (18, 27, 30, 32, 33). In this paper, we will provide the results from one such evaluation.

We Can Move (WCM) began its implementation in April 2018. Through a systems approach, it aimed to influence the physical activity levels across the Gloucestershire population, with a specific focus on populations with very low levels of activity (less than 30 minutes per week). Active Gloucestershire is a voluntary sector organisation that co-ordinated WCM, otherwise known as the 'backbone organisation' (34). They worked with a wide variety of community groups, organisations and system leaders from multiple sectors to overcome the barriers facing individuals (e.g. opportunities for physical activity), communities (e.g. the quality of our physical and social environments), and the wider system (e.g. the policies and people that make the system work as it does). It was anticipated that WCM would not only benefit the physical activity levels in Gloucestershire, but many other associated agendas such as climate change, overweight and obesity, and mental health. Box 1 provides further details about WCM.

In April 2019, Active Gloucestershire commissioned the National Institute for Health Research Applied Research Collaboration West (NIHR ARC West) to evaluate WCM. This paper will: a) describe the mechanisms of action when implementing WCM, b) highlight the barriers to WCM being implemented, c) illustrate key contextual factors that influenced its implementation, and d) demonstrate the range of impacts associated with WCM being implemented. The WCM programme continued beyond the evaluation period.

For peer review only

Box 1: Description of We Can Move

About WCM: In 2017, Active Gloucestershire secured funding to research alternative approaches to improving the population levels of physical activity. During this time, they developed a *Theory of Change* for a new approach, initially named "Gloucestershire Moves". The Theory of Change included systems science, behaviour change principles, and social movement building.

During 2020, the Gloucestershire Moves changed its name to "We Can Move".

The vision for WCM was to inspire, connect, and enable individuals, communities and organisations to help the least active, move more.

It hoped to achieve this by engaging a wide variety of community groups, organisations and system leaders to remove the barriers facing individuals (e.g. the opportunities for physical activity), communities (e.g. the quality of our physical and social environments), and the wider system (e.g. the policies and people that make the system work as it does).

The Theory of Change was initially tested on the Falls Prevention Initiative and the Community-Based Initiative (the two case studies being evaluated). The Theory evolved slightly throughout this piloting, before being phased into all of Active Gloucestershire's work between 2018 and 2021.

WCM cost an estimated £3,000,000 to implement between April 2018 and April 2021.

Further information is available in Nobles *et al.* (35).

About Active Gloucestershire: Active Gloucestershire are a small voluntary sector organisation funded through national and local grants, local public sector organisations, and charitable donations. Within WCM, Active Gloucestershire's role was to facilitate this approach – also known as the backbone organisation. Prior to WCM, Active Gloucestershire's role was to deliver physical activity sessions and other types of interventions to the public. This often meant working with those who were already physically active.

Setting: WCM was implemented in Gloucestershire, England. Population of approx. 640,000 people. Demographic is aging, 95% white British, and has areas of high deprivation. An estimated 130,000 adults do less than 30 minutes physical activity per week, and 52.7% of children doing less than six hours of activity per week.

2. Methods

2.1 Study design

This study draws on the data from a multi-methods evaluation of the WCM programme. Further details of the whole evaluation are available elsewhere (35). Here, we will report on the findings from 31 stakeholder interviews and 15 Ripple Effects Mapping (REM) outputs (36). This work was underpinned by realist evaluation principles (37), seeking to understand the processes by which the programme operates, the contextual factors which influence its implementation and outcomes, and the intended and unintended outcomes associated with WCM. We applied these principles to help make sense of the complexity surrounding the programme. Further details related to the methods are available in Online Supplement I (COREQ Checklist).

2.2 Case studies

Three aspects of WCM were identified to create case studies which would help to explore how the Theory of Change (see Box 1) was implemented in practice, and moreover, how each case study contributed towards the WCM programme. These case studies provided artificial and pragmatic boundaries around a complex, messy programme that would otherwise be unwieldy to evaluate within the resource constraints.

Two case studies focused on smaller projects situated within WCM (falls prevention initiative [FPI] and a community-based initiative [CBI]) and the last explored, more broadly, how WCM engaged with organisations and wider stakeholders around physical activity (Table 1). Each case study was identified

For peer review only - http://bmjopen.bmj.com/site/about/guidelines.xhtml

and agreed upon through discussions between the research team and Active Gloucestershire. An adaptive evaluation framework was applied to each case, meaning that different methods could be applied as needed to best answer the evaluation questions. At the same time, some methods, such as semi-structured stakeholder interviews and REM, were applied across all studies to ensure consistency in the type of data collected and insights generated.

Table 1: Case descriptions

Case 1: Falls Prevention Initiative	Case 2: Community-based initiative	Case 3: WCM movement building
**Background**: Gloucestershire has an aging population. Older adults are at a greater risk of falling and being admitted to hospital due to falling.<br><br>**About**: A healthcare organisation funded Active Gloucestershire to develop a falls prevention initiative for the county, with the stated outcome of reducing falls admissions to the Accident and Emergency department.<br><br>Active Gloucestershire worked with community members and professionals to design and develop Fall-Proof. Behaviour change techniques were used to design a behaviourally informed intervention.<br><br>Fall-Proof aimed to increase older adults' awareness of falls risk, to encourage strength and balance exercises at home, and to increase referrals to community-based strength and balance classes.	**Background**: Barton and Tredworth is a small, culturally diverse, deprived area in Gloucester.<br><br>**About**: Active Gloucestershire wanted to start working in a hyper-local manner that responds to, and works with, residents.<br><br>As part of WCM, Active Gloucestershire received funding from a national organisation to work with the local community, including a group of local women, in Barton and Tredworth. These women wanted to create opportunities for other women, particularly Muslim women, to participate in physical activity.<br><br>This group organised many opportunities for physical activity, linked in with local organisations, and championed physical activity in their communities. Active Gloucestershire facilitated the group.	**Background**: Social movement building is a core component of the WCM Theory of Change. It provides a framework for stakeholders and organisations to integrate into, and take ownership of, WCM.<br><br>**About**: WCM seeks to work with multiple organisations and sectors to make Gloucestershire a more conducive place for physical activity. WCM encourages stakeholders to see their place in the system, to see how they could contribute to making physical activity the norm, and to see that many different agendas would benefit from improved collective working.<br><br>Active Gloucestershire supported and facilitated WCM's implementation, alongside providing opportunities for organisations to engage in and support WCM, and engage with each other.

2.3 Data collection

We focus here on the data gathered using semi-structured interviews and REM. We planned to recruit 10 stakeholders per case study to participate in a semi-structured interview (n=30 total). Prospective stakeholders were purposefully sampled to gather a diverse range of perspectives via three main channels: a) through conversations with the WCM implementation team; b) using the REM outputs; and c) using data gleaned from an annual stakeholder survey.

A topic guide (Online Supplement II) was used to provide consistency within the stakeholder interviews. It paid attention to how stakeholders were involved in WCM, key contextual factors, and the mechanisms of – and barriers to – implementation, and the perceived impacts associated with WCM. Probing questions were then tailored to each interviewee based on content discussed and the REM output which they may be associated with (further information in Table 2). Interviews were

For peer review only - http://bmjopen.bmj.com/site/about/guidelines.xhtml

conducted by the same researcher (JN) between May 2020 and January 2021 using online videoconferencing platforms. Interviews typically lasted 30 - 60 minutes.

Ripple Effects Mapping is a participatory, qualitative method for understanding and visualising the wider intended and unintended impacts of a project or programme. We refined the original in-depth REM technique from Chazdon *et al.* (38) so that it captures the temporal aspects of systems change efforts (36). Data were gathered via workshops and were led by an experienced facilitator (JN). Members of the implementation team were invited to attend these workshops. The facilitator guided participants through a series of activities (Table 2) that led to the creation of a visual REM output which documented the actions and activities undertaken, along a timeline, and their associated initial and wider impacts.

The first REM workshops (December 2019) were conducted face-to-face with the whole WCM implementation and programme team, lasting 120-150 minutes. Due to COVID-19 restrictions, all follow up workshops (with smaller project teams) were carried out online using online videoconferencing platforms. Follow-up workshops (60-90 minutes) aimed to update previous REM outputs with new actions, activities, and impacts that occurred throughout the last 3-4 months. Data were recorded in a paper-based format in the face-to-face sessions, and data from subsequent follow-up workshops were entered into Vensim PLE, and with permission, conversations were recorded directly through online videoconferencing platforms. Further details on how we applied REM in the WCM evaluation is available in a methodological paper (36). See Figure 1 for a simplified and illustrative REM output.

Table 2: Summary of REM stages (36)

REM Stages	Description
1. Introduction to REM	The facilitator gave a brief presentation to introduce the REM method. This included background to the method, the limitations of traditional evaluation methods, and an outline of the approach used in the REM workshops. Not required in follow-up workshops.<br>*Time allowed*: 10-15 minutes
2. Peer/team-based discussions	Participants split into pairs or small project groups and had conversations about the work they are involved in, what they are proud of, what has gone well within their work, and what has been challenging. These discussions were guided by principles from Appreciative Inquiry (36, 39). Conversations served as a warm-up to the main activity.<br>*Time allowed*: 10-20 minutes
3. Mapping the impacts	Each project group was provided with a large piece of paper that had a timeline placed longitudinally across it. The timeline spanned from the beginning of WCM (April 2018) to the present time (December 2019). Participants were asked to think about the main activities or actions that were carried out throughout this timeframe, and to write these onto the map at the time which they occurred. They were then asked to map out the impacts associated with the respective activities. Arrows were draw between activities and impacts to denote the relationship. Participants were encouraged then to map any additional "ripples" that manifest

For peer review only - http://bmjopen.bmj.com/site/about/guidelines.xhtml

		following this activity and / or impact. The facilitator assisted groups where needed. During this time, groups were able to work on REM outputs associated with two to three projects / areas of work.<br>*Time allowed*: 60-90 minutes.
4.	Reflecting on the impacts	Part way through the previous stage, the facilitator asked participants to consider additional questions when mapping. These included: a) identifying intended and unintended impacts, b) stating which organisations / stakeholders / groups were involved or affected, c) financial implications, d) what else may have contributed to impacts occurring, and e) how these activities / impacts link with wider WCM work. Recurring trends may also have been apparent in their outputs. When organisations / stakeholders / groups were identified, these provided the evaluation team with potential interview participants.<br>*Time allowed*: Concurrent to stage 3.
5.	Most and least significant changes	Once most of the actions, activities and impacts are mapped out, the facilitator then asked the groups to identify what they considered to be the most- and least-significant changes in their output. Groups had time to discuss what may have contributed to these changes occurring as they did, and whether in retrospect, they would do anything differently. Data were not recorded for evaluative purposes at this stage.<br>*Time allowed*: 10 minutes
6.	Group feedback and learning	The final part of the workshop provided time for participants to feedback to the group the main aspects of their work that was mapped, what their reflections on the map were, and what they had learnt from the REM process. The facilitator guided this discussion.<br>*Time allowed*: Remaining time.

2.4 Data analysis

Interview recordings were transcribed verbatim and uploaded into NVivo 12 for data management. A framework analysis approach was applied to all qualitative data within the WCM data set (40), which included the data from interviews and REM. The initial framework was devised around core features of interest, including contextual factors, mechanisms of implementation and impact, and intended and unintended outcomes. Data pertaining to the three case studies were analysed independently, so that similarities and differences could be drawn out. Additional content analyses (41) were used to quantify certain aspects of the REM outputs (e.g. number of organisations involved, number of policies influenced etc…). More information regarding the analysis of REM outputs is available in a methodological paper (36). All data were analysed by the lead researcher (JN) and supported by a second researcher (CFox). To increase the dependability of the findings, a researcher (JN) had regular discussions with the implementation team and the wider stakeholders to ensure preliminary findings resonated with their experiences. Findings (not data) were triangulated from the interviews and REM for the purpose of this paper.

[INSERT FIGURE 1 HERE]

2.5 Patient and public involvement

For peer review only

For peer review only - http://bmjopen.bmj.com/site/about/guidelines.xhtml

No patient and public involvement.

3. Results

Thirty-one interviews were conducted across the three case studies. Interviewees represented local government authorities, health care services and commissioning, voluntary and community sector organisations, local elected members, community residents, and Active Gloucestershire staff. Fifteen REM outputs were created with the input of 25 participants (*n*=17 implementation staff, *n*=8 community residents involved in the CBI). Seven outputs were updated over the course of a year (further information available in Nobles *et al.* (36)). The final themes are presented under five key headings: 1) reasons for stakeholder involvement; 2) mechanisms of implementation and impact; 3) barriers to implementation; 4) contextual factors; and 5) impacts and outcomes (Table 3). Text in **bold** below represents a sub-theme.

We have developed a white-water rafting analogy to help explain, and interconnect, many of the findings below. The analogy illustrates how an approach like WCM (represented by the raft) requires a guide (i.e. Active Gloucestershire), a crew (i.e. wider stakeholders), and the ability to negotiate the dynamic nature of the river (i.e. the broader context and circumstances which influence implementation).

For peer review only

For peer review only - http://bmjopen.bmj.com/site/about/guidelines.xhtml

Table 3: Framework analysis

	Case 1: Falls Prevention Initiative (FPI)[a]	Case 2: Community-based Initiative (CBI)[b]	Case 3: WCM movement building[c]
**1. Reasons for involvement**	• Project aligned with, and could help, organisational priorities.<br>• Belief in the project and the associated benefits.<br>• Opportunity to network and share learning across organisations.<br>• Perceived as a "quick win".	• Experienced, or belief in, benefits of physical activity.<br>• Want to increase physical activity opportunities and levels.<br>• Believe in the wider benefits to society and the community (i.e. "not just about physical activity").<br>• Opportunity to learn about new approaches.<br>• Live in the local area.	• Shared priorities, passions and values.<br>• Personal, organisational or sectoral benefits from engagement.<br>• Contractual obligations.
**2. Mechanisms of implementation and impact**	• Backbone role:<br>○ Links with community.<br>○ Signposting to materials.<br>○ Marketing expertise.<br>○ Bringing people together.<br>• Key stakeholder involvement in design of the FPI.<br>• Capitalise on pre-existing relationships.<br>• Dissemination via influential stakeholders and residents.<br>• Formal conduits between organisations.<br>• Behaviour Change Wheel approach to intervention design.<br>• COVID-19 causing statutory organisations to consider alternative ways of working.	• Backbone role:<br>○ Pivotal mechanism.<br>○ Facilitative.<br>○ Provide knowledge, expertise, and credibility.<br>○ "A way of working".<br>• Steering group role:<br>○ Understand local needs and wants.<br>○ Tailored, non-judgemental meetings.<br>○ Proactive and relatable community members.<br>○ Central hub to nurture ideas.<br>○ Disseminate information to community about physical activity and related opportunities.<br>○ Champion physical activity for community.<br>• Engage the right people and organisations.<br>• Create a collective and shared vision.<br>• Establish effective communication channels between stakeholders.<br>• Understand root causes of low physical activity, driven by data and insight.<br>• "Go with the flow".<br>• Keep it local & draw on community assets.<br>• Selling physical activity through persuasive and creative communications.	• Backbone role:<br>○ Facilitating (and disrupting) the system.<br>○ "Joining the dots" - fostering new relationships.<br>○ Linking in with, and establishing, professional networks.<br>○ Influencing the system and system leaders.<br>○ Imparting knowledge and expertise.<br>○ Designing and delivering projects.<br>○ Supporting other organisations.<br>○ Fiducial responsibilities.<br>○ Champion WCM and physical activity.<br>• Adaptive and strong governance for WCM.<br>• Developing strong foundations for WCM:<br>○ Passion of Active Gloucestershire team.<br>○ Informal meetings.<br>○ Council and senior leadership support.<br>○ Alignment with strategic priorities in the county.<br>○ Funding in place.<br>• Working with the right people who have a shared vision, agenda and purpose.<br>• Focus on building strong relationships between people and organisations.<br>• Events and networks can initiate and catalyse relationship building.<br>• Develop conduits between organisations and WCM.<br>• Ability of WCM to "read the river" – timing and conditions.<br>• Celebrating success.<br>• COVID-19 led to new opportunities, funding, and partnerships.
**3. Barriers to implementation**	• Clarity on roles, responsibilities, and involvement of wider stakeholders.<br>• Communication between backbone organisation and wider stakeholders.	• Overcoming bureaucracy, red tape, and administrative challenges.<br>• Monitoring "success" of CBI's.	• Stakeholder understanding, communications, and perceptions of WCM.<br>• Clashing of mindsets.<br>• (Continued) Engagement in a social movement.

For peer review only - http://bmjopen.bmj.com/site/about/guidelines.xhtml

For peer review only

	• "Demonstrating impact" - evaluation, measurement, and monitoring of complex projects.<br>• Managing conflicting stakeholder tensions, mindsets, and ways of working.<br>• COVID-19 impact on delivery and participation.<br>• Linking the FPI to WCM.<br>• Backbone organisation capacity.<br>• Length of time to design intervention.	• At odds with transactional mindsets of organisations and senior leadership.<br>• Limited stakeholder engagement.<br>• Acknowledge challenges within a steering group (format, aims, opposing views).	• Inconsistent preferences surrounding evaluation.<br>• COVID-19 prevented progress and collaboration across WCM.<br>• COVID-19 impact on stakeholder engagement, and prioritisation of WCM by organisations.
**4. Contextual factors**	• Institutional transactional ways of working.<br>• History of local falls prevention work.<br>• Organisational strengths of Active Gloucestershire.<br>• Active Gloucestershire going through transition period.	• Active Gloucestershire as a trusted, well-connected, and established organisation.<br>• Established Voluntary and Community Sector and history of community building.<br>• Mindset shift occurring in some organisations.<br>• Negative perception towards previous approaches - "[locality was] a picked-on area".<br>• Varied demographic of local community.<br>• Cultural norms against physical activity.	• Active Gloucestershire well-connected, respected, and knowledgeable organisation.<br>• "WCM pushing on an open door".<br>• Demographic profile of Gloucestershire.<br>• COVID-19 implications.
**5. Impacts and outcomes**	• Awareness, use and uptake of the FPI work.<br>• Quality of the FPI materials.<br>• Stakeholders feeling part of something bigger.<br>• Wider engagement in FPI materials (locally and nationally).<br>• Limited impact on referrals to strength and balance providers.	• Perceived increases in community physical activity.<br>• Improved / changed physical activity infrastructure and provision.<br>• Enhanced community engagement, cohesion, and empowerment.<br>• Growth, reach and self-organisation of work.<br>• Influence on, and buy-in from, wider sectors.<br>• New money and funds generated.<br>• Steering group:<br>○ Group ownership changed over time.<br>○ Buy-in from local councillors.<br>○ Feel part of something bigger.<br>○ Feel empowered and accomplished.	• Successful buy-in and support for WCM.<br>• Mindset shifts within organisations and senior leaders.<br>• "Linking up the system" – new relationships created.<br>• "Systems change happening in pockets".<br>• Beneficial impacts and influence on communities, organisations and sectors.<br>• Uptake of physical activity and interventions.<br>• New money and funds generated.<br>• New projects established.<br>• Other areas adopting / interested in WCM.<br>• Supporting, benefiting and aligning with other strategic agendas.

[a] **FPI data collection**: 12 stakeholder interviews, one REM output (created over four iterations).

[b] **CBI data collection**: 10 stakeholder interviews, one REM output (created over five iterations).

[c] **WCM movement building data collection**: Nine stakeholder interviews, 13 REM outputs (five created over multiple iterations).

For peer review only

3.1 Reasons for involvement

Stakeholders frequently had multiple reasons for getting involved in WCM. Across the three cases, stakeholders believed in the **co-benefits** associated with WCM and physical activity (e.g. mental health, social connections, health inequalities), which could be realised by individuals, communities, organisations, and wider sectors (e.g. Transport or Social Care). Many also noted that WCM aligned with the **strategic priorities of their own organisation**, and so their involvement in WCM would be mutually beneficial. For example, one stakeholder highlighted how WCM could benefit a council agenda around adverse childhood experiences (ACEs):

> *"ACEs is one of the sort of Health and Wellbeing Board priorities and it has been a priority since 2018. Part of that and the research around ACEs was around, we know that you can build resilience through regular sports participation and we're using the five ways to wellbeing as our sort of resilience building tool." – Participant 2, Case 3, County council public health*

Other reasons were more specific to individual cases. Some bought into the FPI because they believed it would bring about "quick-wins" – most notably, reductions in the number of people being admitted to hospital because of falling. On the other hand, in the CBI, several community residents became involved because they lived locally and wanted to benefit the community, whilst others wanted to learn about the WCM Theory of Change (Box 1).

3.2 Mechanisms of implementation and impact

Our white-water rafting analogy is particularly useful here to present the three main mechanistic themes. These include: 1) the role of the guide (i.e. the backbone organisation), 2) developing the right crew (i.e. wider stakeholders), and 3) the ability of WCM to "read the river" (i.e. local context and circumstances). These factors are interconnected and interdependent, as explained below, using the white-water rafting analogy.

A fundamental **role of a guide** is to steer the raft by talking to, motivating, and inspiring the crew. In WCM, Active Gloucestershire held the role of the guide, also referred to as the "backbone organisation". Whilst the backbone role varied across the three case studies, its importance was widely acknowledged by participants. For example, in the CBI (Case 2), the backbone role was held by one staff member of Active Gloucestershire. Interviewees stated that they were pivotal in that they facilitated relationships between stakeholders, that they provided knowledge and expertise (about physical activity and about local networks of stakeholders and organisations), and that their role – and the way in which they worked – was "*more than a job*" (Participant 6, Case 1, Community member). In the context of WCM (Case 3), the backbone role was held by Active Gloucestershire as an

For peer review only - http://bmjopen.bmj.com/site/about/guidelines.xhtml

organisation. Active Gloucestershire were seen to be centrally involved in facilitating how the physical activity system was organised – from the stakeholder networks to the way in which funding was distributed within the sector. This was referred to as a "*behind the scenes*" role (Participant 7, Case 3, Local third sector organisation). Thus, they were believed to have substantial influence given their strong connections to senior leaders across Gloucestershire, further reinforced by their credibility as an organisation. Interviewees also spoke of the intricate knowledge (of people, places, and communities) and extensive expertise (in physical activity, project management, and behaviour change methodology) that Active Gloucestershire held and were able to share with others. A large part of the backbone role was to "*join the dots*" (multiple interviewees references) – to bring people, organisations, and their agendas, together. They did this via formal (e.g. setting up stakeholder networks) and informal (e.g. email introductions) methods. Several interviewees reported that Active Gloucestershire provided in-depth advisory support to their organisation given that they were relatively new to the county and/or work in the Public Health sphere. At their core, Active Gloucestershire were passionately championing, facilitating and co-ordinating WCM.

> *I think that what Active Glos did getting all those people together, was the contacts that they've got and the networks that they've got. They're very different from our health network, so I think that was brilliant. It did make sure that the community voice from a lot of different perspectives remained through those [FPI] planning stages definitely. – Participant 3, Case 1, NHS commissioning*

> *I think it's imperative to have somebody on the ground that knows the local area, can build a relationship with local people, they've got that local knowledge, they can connect like-minded people and just offer that bit of support – Participant 3, Case 2, City council*

To negotiate the turbulence of the river, a raft needs more than a guide, it needs a crew. This is where the implementation and success of WCM was largely hinged on its engagement with wider stakeholders. Interviewees said that Active Gloucestershire placed a lot of effort on **developing the "right" crew**; the people and organisations to work with as part of WCM. The "right" people and organisations typically comprised those whose *vision* (i.e. how they work and what they are aspiring to) aligned with that of WCM, and whose *agenda* (i.e. the things that are important /prioritised within their organisation) would benefit from the work of WCM. These two elements, vision and agenda, enabled wider stakeholders to see value in WCM and to better see their role in WCM. Furthermore, interviewees spoke about the personality and behavioural characteristics (or "mindset" – i.e. way of thinking about problems and actions) of wider stakeholders who were influential within WCM. This included an ability to build strong relationships, to look and work beyond their own organisational priorities (and thus acknowledge that they work within a system rather than silos), and to feel

For peer review only - http://bmjopen.bmj.com/site/about/guidelines.xhtml

comfortable working – or attempting to work – in a different, and perhaps more collaborative and equal, manner. These crew members often acted as conduits for information about WCM to flow through to their senior management, therefore working as a mechanism to increase organisational buy-in to WCM.

> *I have always been that new way, I've always done this sort of way, I always think connecting the dots is much better, more effective so I've never worked in that sort of old way and that silo approach I guess. So I think there might be a bit of challenge around how they work and you know bringing people – great, get everyone in a room, get everyone interested but then you might get some people that would just automatically flip back to, right what's on priorities, what's on the agenda I've got to do today. – Participant 2, Case 3, County council public health*

To identify the "right" people, Active Gloucestershire staff invested substantial time in attending and organising networks (via REM analysis, at least 23 networks were attended or organised). These networks often led to serendipitous outcomes; for example, Active Gloucestershire staff met new people and organisations, organised meetings outside of the networks, which subsequently led to unplanned joint working that would benefit the overall implementation of WCM (and the work of the other organisation). We noted, through the REM analysis, that 82 organisations had been engaged in WCM throughout the data collection (April 2019-January 2021), and there were many examples of these unanticipated partnerships in the dataset. Most of the work, and relationships developed, in the CBI (Case 2) came about because of an initial network meeting held by Active Gloucestershire called "Joining the Dots" which was attended by approximately 65 local organisations. However, the REM and interview analyses also suggest that time was needed to develop meaningful, trusting relationships, while the length of time required varied dependent on the stakeholder or organisations engaged with.

With the guide and crew established, the final key mechanism was the ability for WCM to "**read the river**". Interviewees described how WCM (and associated projects) had been able to adapt to local and national contexts and circumstances (see COVID-19 sub-theme below), and how key stakeholders (i.e. guide and crew) could react quickly to these circumstances to benefit the overall approach. For example, this meant being able to a) develop proposals with organisations quickly to secure new funds, b) present evaluative information to funding partners to re-assure them that progress was made, and c) change governance structures to better support the vision of WCM. This need for adaptation was recognised by many of the interviewees. In contrast, the FPI (Case 1) perhaps resembled a more traditional approach to service delivery; it had key performance indicators, it was held accountable by the funding organisation to these indicators and deliverables, and the relationship between funders and the providers was considered somewhat transactional. Here, we saw a clash

For peer review only - http://bmjopen.bmj.com/site/about/guidelines.xhtml

between traditional- and the desired- ways of working within WCM. We Can Move appeared, according to interviewees, to work more effectively when it was able to alter its course in response to the ever-changing, choppy nature of the river (i.e. circumstances and context).

> *It doesn't feel within the [organisation] to… there's not that trust, I don't believe, from the [organisation] for us to adapt the programme because we are seeing a need to change and adapt and I think there's not that trust of, okay, we understand that like there is in some other areas of We Can Move. I think with this [the FPI] you have to justify and evidence every change you make... – Participant 8, Case 1, FPI implementation*

> *It's all about the relationships isn't it and you can't – the organisations you might think you are best placed to work with might not want to work with you or you might not have the relationship established. So yes you kind of have to build that don't you, in the short term it might mean doing different things or working with different partners, because as you say that's where the appetite is and the energy is. – Participant 8, Case 2, National physical activity organisation*

The **COVID-19 pandemic** both foregrounded and bolstered the ability of WCM to adapt to changing contexts. Whilst creating many challenges for the implementation of WCM, COVID-19 was also a catalyst in enabling or necessitating multi-sectoral, multi-organisational working. This theme was particularly present within the REM analyses. For example, in the FPI (Case 1), the educational materials were disseminated more broadly than initially anticipated – and through new organisations – due to the requirement for many older adults to isolate in the early waves of the pandemic. More broadly across WCM (Case 3), the impact of COVID-19 meant that many organisations had to de-prioritise their involvement in WCM to remain operational. This subsequently meant that WCM had to alter its priorities which it was able to do in part because of its adaptive governance structures (i.e. the ways in which other organisations become involved). As new money became available across the system from central government and other national funding agencies, Active Gloucestershire, due to their credibility as an organisation and centrality within the local networks, were able to bring stakeholders together from multiple organisations to capitalise on these funding streams to support the health – and wider needs - of local populations. In turn, this then led to new areas of work, new relationships, and wider impacts of WCM across the system.

> *I guess COVID and needing to work in a new way just made people look up a bit [i.e. look outside of their organisational silo] because there was a need. Previously you had a service and then suddenly you didn't, so there was a gap which you couldn't fill with anything traditional. Why would you look up when you've got a working service, essentially? But when that need came, people were like, 'Okay, so what can we do differently?' and then that's when those links –*

For peer review only - http://bmjopen.bmj.com/site/about/guidelines.xhtml

people were like, 'Okay, I'll ask if he's got any ideas' or… - Participant 6, Case 1, NHS commissioning

3.3 Barriers to implementation

Four main barriers to the implementation of WCM were identified. These include challenges related to evaluation, issues surrounding opposing mindsets within WCM, misaligned understanding and expectations of WCM, and the negative implications of COVID-19.

The **evaluation** of WCM, and associated projects (e.g. the FPI and CBI), was problematic for several reasons. Across the three case studies, there were inconsistent expectations around the purpose and role of the evaluation across WCM. For example, in Case 2, several interviewees stated that it was difficult to measure and capture the unanticipated impacts of a community-driven initiative (that did not have a predetermined project plan). In Case 1, interviewees disagreed on whether it was important to focus on the "hard numbers" associated with the FPI to determine the success of the project. For some, these numbers (i.e. reduction in number of people being admitted to hospital due to a fall) were the most important "success" measure, but for others, especially those closest to the implementation, believed that it was not feasible to monitor this or attribute change to the intervention directly. These issues around attribution of effect were also noted amongst the wider WCM stakeholders (i.e. Case 3).

They [senior council leaders] were literally grilling the community builders and I don't think I was meant to stand up, I was just in the gallery [of the Town Hall] viewing the whole thing and I just said, 'Look, can I speak a minute?' I said, 'At the end of the day this isn't something that you can just see happening overnight, you have to wait.' It's taken however long for me to get to where I am now, 'cause I'm doing, I suppose you could say, unofficial community building, but as a volunteer, I suppose, but it takes time. If I expect everything to happen overnight, I'll just give up and won't bother and so I think back to me, this is something that you can't just assess overnight and you've got your answers, you have to wait to see the positives that come out of it. – Participant 4, Case 2, Community member

This is closely entwined with the next barrier, a **clash of mindsets** within WCM. In part, it appeared that the expectations surrounding the evaluation were linked to the mindset and ethos of the individual, organisation and/or sector. We Can Move was advocating for an alternative way of working across the system, meaning that multiple organisations are involved in the implementation and adaptation of WCM over time. However, each organisation coming aboard WCM had their own ways of working, and in particular, some organisations adopted transactional ways of working. This meant that these organisations were typically accustomed to commissioning services, setting key

For peer review only

performance indicators (thus linked to evaluation issues above), having regular performance update meetings, and therefore having a hierarchical relationship between commissioner and provider. By and large, this way of working was at odds with WCMs – referred to as "*the old and new ways of working*" (Participant 5, Case 3, NHS commissioning). This picture is nuanced though. In both the FPI (Case 1) and WCM (Case 3) interviews, several interviewees stated that whilst their own preferred way of working aligned with WCM, they found themselves working in organisations that were predominantly, and traditionally, transactional. This created significant challenges for these individuals – who were often the conduits and brokers between WCM and their organisation – as they held a mediating role, trying to re-iterate the purpose of WCM to their organisational leaders whilst simultaneously trying to demonstrate the value of WCM and reassure leaders that their investment in WCM was useful.

Misaligned understandings and expectations surrounding WCM were another common barrier mentioned in Cases 1 and 3. Several interviewees, especially those who were more peripheral to WCM, stated that they did not understand what WCM was and how it differed from Active Gloucestershire. We Can Move was seen as intangible by many of these stakeholders, and in part, this was due to what they perceived as complicated messaging. The premise of WCM was not consistently communicated, or understood, by those involved and this contributed to further misunderstanding and confusion. Similarly, if interviewees were predominantly involved in a project (e.g. FPI or CBI) rather than WCM more broadly, they often struggled to see how these particular projects linked with the wider work of WCM. And so, when asked what their role was within WCM, these interviewees either a) did not see themselves having a role, or b) did not know what was expected of them. Whilst one of the strengths of Active Gloucestershire, noted by interviewees, was their communication with wider stakeholders, this was not consistent.

The last barrier was that of **COVID-19**. It is important to note that most of the data collection for the second case study, the CBI, was undertaken before the pandemic began, and so there was very little data on this in the data set. In the other two cases, COVID-19 meant that much of the planned implementation work was paused or cancelled, and WCM and the FPI were required to adapt in response to the imposed restrictions (see mechanisms section). The primary reasons for this stagnation were that wider stakeholders reverted back to their silos because their organisational priorities changes (i.e. the need to "*survive*" – Participant 7, Case 3, Local third sector organisation) and many organisations had to furlough staff. This prevented progress being made between stakeholders who were involved in WCM before the pandemic began.

For peer review only - http://bmjopen.bmj.com/site/about/guidelines.xhtml

It's just been such a rubbish year and I think that that has overshadowed and become even more of an obstacle to getting stuff going because you've got the people that are shielding for themselves or others [from COVID-19], the low mood increase, lack of motivation, the disinterest in doing anything because things almost seem pointless… You've got to understand it and it's been impossible, I would say, to get things as good as they could be. – Participant 6, Case 3, Local third sector organisation

3.4 Contextual factors

Contextual factors are those which are not part of the intervention, or were already established pre-implementation, but affect its subsequent implementation and impact (42, 43). Interviewees often described the **organisational strengths of Active Gloucestershire**; the organisation was said to be well-connected, respected and a reliable source of knowledge within the county. In short, Active Gloucestershire were already well placed to guide the WCM raft, to be the backbone organisation. That is, the organisation that facilitate, and are accountable for, the implementation (and adaptation) of WCM.

"The result of one of those conversations [with senior leaders from the county] was the head of commissioning said, 'We need your people off furlough,' and that led to us working over the summer holidays with children that were in social care, have got PTSD, all these things… We would have found our way to that table, but it would have taken a lot of leg work, whereas Active Gloucestershire have got these inroads to these various different partners and have been great at keeping that door open for charities like us." - Participant 8, Case 3, Local third sector organisation

Stakeholders also suggested that WCM was **pushing on a slightly open door**. The council, and several national and local funders, were becoming more receptive to systems approaches. There was already a history of falls prevention work in the county (Case 1), a growing interest and application of place-based and asset-based community development (Case 2), and a rich and cohesive voluntary sector within the CBI area (Case 2). Systems approaches have also been adopted in Gloucestershire to other issues such as obesity and ACEs. That said, some organisations involved in Case 1 were said to work in a transactional manner whereby Active Gloucestershire are accountable to a commissioner. Organisational engagement in WCM was influenced in part by their institutionalised ways of working. Thus, linking back to the analogy, Active Gloucestershire had prior knowledge of some of the river conditions before commencing WCM. They also had several crew members, or at least their respective organisations, warmed up and ready to go.

"It's almost like you want to put a heat map over the county, or a magnet, and there are some particular individuals that are really, really interested in systems change. They can see the

For peer review only - http://bmjopen.bmj.com/site/about/guidelines.xhtml

> *strategic benefit of it. However, many of those individuals are stuck in traditional institutions and therefore really struggle to innovate, so they will try and be supportive but there'll be certain hoops that they have to jump through or political issues that they're encountering" – Participant 3, Case 3, Local third sector organisation*

3.5 Impacts and outcomes

We derived four main types of impact from the analysis: 1) physical activity-related impacts, 2) relational impacts, 3) sectoral impacts, and 4) null or negative impacts. It is important to note here that the **physical activity-related impacts** were not objectively measured, but rather they were perceived changes in physical activity noted by the interviewees or REM participants. Interviewees – especially in relation to Case 2 – suggested that the opportunities for physical activity amongst Black, Asian and Minority Ethnic (BAME) women had improved. This was also reported in relation to the older adults (Case 1) who received the educational materials from the FPI. More broadly though, in Case 3 and through the REM analysis, we identified many instances where physical activity, for individuals, communities, and populations, may have been influenced.

> *They had over 100 women get involved cross all the different activities, and mostly BAME women who wouldn't engage, or who aren't very well connected. So, when the different women come in, they have carried on as well. Over 100 women. That's not bad when you started off with just 12 women who wouldn't engage with other communities. – Participant 1, Case 2, City council*

The main impacts however, as per this analysis, were **relational** and **sectoral**. There was said to be improved cohesion and collaboration between stakeholders involved in, or on the periphery of, WCM, and many examples were given. As previously noted, we found that at least 82 organisations were involved in WCM and that 23 networks were attended or organised. Being part of WCM enabled stakeholders to feel like they were part of something bigger, an initiative or way of working that went beyond their own organisation. This was supported by the generation and dissemination of new funding via WCM – enabling organisations to come together. It was through these new or improved relationships that we saw cross-sectoral impacts; for example, the work of WCM aligned with and supported the work being undertaken on mental health, air quality, tackling inequality, and town planning (among others). And many commented that their own mindset (i.e. a way of thinking) and that of their senior leaders had started to change due, in part, to their involvement in WCM. Lastly, through the REM, we found that WCM and physical activity had been integrated into a minimum of six policies across the county, ranging from the Health and Wellbeing Strategy (county wide) to City Planning policies (City wide) to Transport Planning policies (City wide).

For peer review only - http://bmjopen.bmj.com/site/about/guidelines.xhtml

Like I say, there's other people that you see and other ladies and it's finding out what they're doing as well. It's doing things together as a group. They're not only like – it's not to be said, we're going to [steering group member A] and nobody knew about [steering group member B]. It's given [steering group member C] the opportunity to get to know the people in the community in this area. – Participant 9, Case 2, Community member

There were some **negative or null impacts** reported too. Some interviewees stated that there had not been any beneficial impacts on them or their organisation, and that this was due to a lack of engagement or an inability to engage due to COVID-19. This meant that some of the organisations which were involved in WCM were still working, largely, in silos. Other null impacts were more specific to individual case studies, for example, some interviewees said that the FPI had not improved the number of older adults being referred to strength and balance classes.

I thought after the [systems] mapping exercise it was so nice to bring all those organisations together, really beneficial. We had some really positive conversations and I was really hoping there would be some follow-up to that so that I can then start to work with more organisations and look at how we fit as part of this wider approach and not just in silo. I think, for me, that's what We Can Move is supposed to be. It's to bring everyone together and, yeah, create a system change, whereas we're still just cracking on as we're doing. – Participant 7, Case 3, Local third sector organisation

4. Discussion

There is currently limited evidence available regarding the implementation of systems approaches in the field of physical activity and across public health more broadly (27). This paper highlights the key contextual and mechanistic factors that were associated with the implementation of WCM in Gloucestershire. It also illuminates the types of impact experienced within the first three years of implementation, but notes that there were several barriers which influenced the implementation, and thus, the impact of WCM. Given that there is a paucity of research, this paper provides an important, novel, and timely contribution to the evidence base, with several implications for future practice and evaluation.

4.1 What's needed to start and maintain a systems approach?

Our study identified several important factors to consider when initiating and maintain a systems approach. Understanding, and talking to, the motivations of the wider stakeholders across the system was imperative to kick-starting this work. Active Gloucestershire already had strong relationships across the county, but nevertheless, they had to draw people into WCM. This meant understanding

For peer review only - http://bmjopen.bmj.com/site/about/guidelines.xhtml

the strategic priorities of other stakeholders and organisations, and in doing so, talking to the shared benefits that could be realised through their involvement in WCM. For example, many organisations were looking to do more partnership work across the county. Active Gloucestershire were able to highlight that a core benefit from engagement in WCM would be that organisations are encouraged to work more closely together, to achieve greater impact, and improve the use of scarce resources.

The initial and successful engagement of stakeholders helped WCM to develop the "right" crew (linked to the rafting analogy). Understanding the initial motivations of the wider stakeholders, and speaking to the shared benefits, brought them aboard. However, once aboard, the role of Active Gloucestershire (as the guide of the raft) was to help stakeholders see the bigger picture – to recognise that their own work and priorities are intertwined with others – and to consider the ways in which they could work together. Formal networking events and meetings, as well as informal relationship brokering, were methods used by Active Gloucestershire to encourage collaboration and a shared vision between stakeholders. These are not new phenomena and are deemed by many to be core ingredients of a systems approach and primary outcomes of systems or adaptive leadership (26, 27, 34, 44-48). In effect, Active Gloucestershire were developing the crew and encouraging them to paddle together towards a common goal.

Adaptation is one of the main characteristics of a complex adaptive system (49), and therefore has to be accounted for and anticipated when implementing a systems approach. The impact of COVID-19 caused substantial and rapid adaptations in the local, national, and international systems. For WCM, it meant that many pre-planned initiatives had to be paused (e.g. engagement with schools), but it also brought about new opportunities for collaborative working which may otherwise not have occurred. Given the flexibility in the design of WCM, it was able to adapt accordingly to these turbulences rather than coming to a halt. It did mean though that some stakeholders, such as the local public health team, became less involved (as they were integral to the COVID-19 response) whilst other organisations, such as local faith groups, became more involved (e.g. through distribution of food and activity packs to the community during COVID-19 lockdown restrictions). We referred to this adaptability of WCM as "reading the river". Donella Meadows refers to this as "dancing with systems" (50).

4.2 What type of impacts may we expect from a systems approach?

Capturing the diversity of impacts arising from a systems approach is difficult (51). Within this evaluation, we brought together two methods (REM and semi-structured interviews) which we found helpful in trying to better understand the anticipated and unanticipated impacts of WCM across the Gloucestershire systems – i.e. the adaptive and emergent nature of the system and of WCM. Ripple

For peer review only - http://bmjopen.bmj.com/site/about/guidelines.xhtml

Effects Mapping allowed us qualitatively to capture, close to real time, many of the activities and impacts associated with WCM over a two-year period. The interviews were then used to gather further information about these impacts, and to learn more about how these impacts may have occurred. Our analysis showed that there were perceived changes in physical activity among particular communities (e.g. Muslim women within an area of Gloucester – Case 2), and changes to the physical activity environment (e.g. changing policies around access to leisure facilities and changes to physical activity opportunities in school). However, as others have found (52), system approaches such as WCM foster new relationships – and cohesion between stakeholders – across the system.

Using the REM, we were also able to demonstrate where WCM benefited other strategic priorities and made intangible impacts, tangible. We noted that WCM was embedded into six policies and had aligned with 22 other agendas (including air quality, town planning, inequalities, and mental health). In the 2019 Lancet commission (23), Swinburn *et al*. refer to such initiatives as double or triple duty actions – those which contribute towards several outcomes at once. Linking back to the initial role of Active Gloucestershire, and perhaps other backbone organisations, we were able to see how the appeal of shared benefits as a way of initially engaging stakeholders can be realised through a systems approach. We also documented less tangible, or softer, impacts through REM which included the changing mindsets of senior leaders within the system, enhanced and more cohesive relationships between partners, and the WCM Theory of Change being considered in other areas of the UK. While the relational and mindset aspects of these intangible outcomes are aspired when implementing a systems approach (22, 26, 44, 51), they are seldom reported on, but here, REM enabled us to surface, capture and analyse these as part of the WCM evaluation.

4.3 What might moderate the implementation and impact of a systems approach?

We highlighted several key factors which influenced the implementation and impact of WCM, and these would likely occur in other areas who are aiming to adopt a systems approach. In line with characteristics of a complex adaptive system (53), it is important for stakeholders to assess the "initial conditions" in which the systems approach is to be implemented (54, 55). Gloucestershire – and many of the organisations within it – was a county which was somewhat primed for WCM; some people (e.g. council staff, NHS staff, Voluntary and Community Sector organisations) were familiar with systems approaches, the associated jargon, and many had already established cross-sectoral relationships. Similarly, Active Gloucestershire were a well-respected and credible organisation. Linking back to the rafting analogy, WCM started out with a sturdy raft, a strong guide, and a forming crew. Some wishing to adopt a systems approach may also have strong foundations to build on, whilst others may not, and

For peer review only - http://bmjopen.bmj.com/site/about/guidelines.xhtml

so prospective implementers and evaluators need to be mindful of the starting point and anticipate what impact this may have on early efforts (42, 56, 57).

Once WCM commenced, other challenges arose which influenced the rate at which it built momentum. A major challenge was a clash of mindsets, between those wishing to implement – or be involved in – a systems approach and those wanting to retain a more traditional service commissioning / delivery hierarchy. This becomes more problematic if these are institutional or sectoral mindsets (e.g. across healthcare) rather than that of a particular individual (e.g. a manager within healthcare). Historically in the UK, and elsewhere, public and population health (which include physical activity) has been delivered using a traditional medical model (18, 58-60). In turn, this means that many issues – such as low levels of physical activity – are treated and prevented akin to other medical issues. Thus, this often leads to the funding, commissioning and provision of individual focused services and programmes – for example, physical activity sessions, health promotion campaigns, weight management programmes etc. These medicalised approaches have been deeply engrained within healthcare and public health, and in part, have contributed to siloed working (61, 62). In this evaluation, despite endorsement of WCM, some stakeholders and organisations appeared uncomfortable with the WCM approach; it meant devolved power, shared responsibility and ownership, less tangible outcomes, and accepting uncertainty. These are not findings unique to this context. Crane *et al.* (62) highlighted this as one of four major challenges facing the sustainment of population health programmes, and Alderwick *et al.* (48) noted it as a significant hurdle facing the collaboration between health care and non-health care organisations. Many see bold, and systems, leadership as a means of working through these challenges (44, 45, 47, 59, 62).

4.4 Future considerations

This research has highlighted several important implications and considerations for those implementing or evaluating systems approaches in Public Health. First is the importance of understanding context. Both implementers and evaluators should spend considerable time understanding the initial conditions within the local systems (e.g. organisations, relationships, key actors, local action and policy, historic socio-political factors etc…). These conditions – as shown in the results – contributed to the initial implementation of WCM. Second is the need for stakeholders to determine what lies within the scope of the evaluation – acknowledging that different people will value different elements of the systems approach. Where possible, stakeholders should seek to establish the evaluation boundaries and foci early and clearly communicate these to all involved stakeholders. Note too that these boundaries and foci may change over time. Building on this, third is that evaluators may wish to examine the different perspectives of wider stakeholders involved in the

For peer review only - http://bmjopen.bmj.com/site/about/guidelines.xhtml

approach. Aim to understand their motivations for engagement, what they know of the approach, how they anticipate it to work, and what impacts and outcomes would be of value to them. This may help ensure that the evaluation is meaningful to a range of actors, not just those who are interested in the primary outcome (i.e. increasing population physical activity). A fourth consideration is to question how principles of systems science can be further embedded in the implementation and evaluation of these approaches. For example, how can evaluations gather more information on the adaptive nature of the system, and of systems change efforts? How can implementers ensure – or enhance the likelihood of – identifying and changing deep-rooted leverage points within the system? How can both parties ensure that the evaluation contributes to the continued learning and refinement of the systems approach? And fifth, as others point out (57, 63), further methodological innovation and publication of similar evaluations are warranted to advance the knowledge base around Public Health-orientated systems approaches.

5. Conclusions

This paper is one of few to report on the evaluation of a physical activity-orientated systems approach. We highlighted several important mechanistic and contextual factors which enabled WCM to function as it did, and moreover, captured – via a novel REM method – a wide range of impacts, both anticipated and unanticipated, that were associated with its implementation. These findings are important for the field. They present an honest and robust account of a wide range of stakeholder perspectives who were engaged in, or affected by, a systems approach. They highlight some of the barriers experienced when implementing the approach, and it is likely that others will come across these too in the future. This paper advances the evidence base and provides the findings from a novel, participatory method of REM which was triangulated with interview findings. Further research is needed to continue building the evidence base in this area.

For peer review only

For peer review only - http://bmjopen.bmj.com/site/about/guidelines.xhtml

Additional information

Funding statement: The evaluation of We Can Move was commissioned by Active Gloucestershire. Some of James Nobles', Russell Jago's, and Sabi Redwood's time was supported by the National Institute for Health Research (NIHR) Applied Research Collaboration West (NIHR ARC West). The views expressed in this article are those of the author(s) and not necessarily those of the NIHR or the Department of Health and Social Care. The Article Processing Charge was funded through NIHR ARC West. Award / grant number: N/A

Competing interests: The authors have no competing interests to declare.

Author contributions: JN, SR, RJ & CFos conceptualised the paper, and JN wrote the first draft of the manuscript. All authors (JN, CFox, AIW, TB, SR, RJ & CFos) provided substantial input into later drafts of the manuscript. All authors refined and discussed subsequent versions of the manuscript before approving the final version.

Data sharing statement: No additional data available.

Ethics approval: Ethics approval was granted by the Faculty of Health Sciences, University of Bristol (*Ref*. 91145) and all participants provided written informed consent.

For peer review only

For peer review only - http://bmjopen.bmj.com/site/about/guidelines.xhtml

References

1. Guthold R, Stevens GA, Riley LM, Bull FC. Global trends in insufficient physical activity among adolescents: a pooled analysis of 298 population-based surveys with 1·6 million participants. **The Lancet Child & Adolescent Health**. 2020;4(1):23-35.
2. Guthold R, Stevens GA, Riley LM, Bull FC. Worldwide trends in insufficient physical activity from 2001 to 2016: a pooled analysis of 358 population-based surveys with 1·9 million participants. **The Lancet Global Health**. 2018;6(10):e1077-e86.
3. Sport England. Active Lives Adult Survey - May 2019/20 Report. London, UK; 2020.
4. U.S. Department of Health and Human Services. 2018 Physical activity guidelines advisory committee scientific report. 2018.
5. Lee IM, Shiroma EJ, Lobelo F, Puska P, Blair SN, Katzmarzyk PT. Effect of physical inactivity on major non-communicable diseases worldwide: an analysis of burden of disease and life expectancy. **The Lancet**. 2012;380(9838):219-29.
6. International Society for Physical Activity and Health (ISPAH). ISPAH's Eight Investments That Work for Physical Activity. 2020.
7. Ding D, Lawson KD, Kolbe-Alexander TL, Finkelstein EA, Katzmarzyk PT, van Mechelen W, et al. The economic burden of physical inactivity: a global analysis of major non-communicable diseases. **The Lancet**. 2016;388(10051):1311-24.
8. Bull FC, Al-Ansari SS, Biddle S, Borodulin K, Buman MP, Cardon G, et al. World Health Organization 2020 guidelines on physical activity and sedentary behaviour. **British Journal of Sports Medicine**. 2020;54(24):1451-62.
9. Jáuregui A, Lambert EV, Panter J, Moore C, Salvo D. Scaling up urban infrastructure for physical activity in the COVID-19 pandemic and beyond. **The Lancet**. 2021;398(10298):370-2.
10. Shilton T, McLaughlin M, Reece L, Chalkley A, Gomersall S, Schipperijn J, et al. ISPAH's Eight Investments That Work for Physical Activity. In: Health ISfPAa, editor. 2020.
11. Rutter H, Cavill N, Bauman AB, F. Systems approaches to global and national physical activity plans. **Bulletin of the World Health Organisation**. 2019;97:162-5.
12. Cavill N, Richardson D, Faghy M, Bussell C, Rutter H. Using system mapping to help plan and implement city-wide action to promote physical activity. **J Public Health Res**. 2020;9(3):1759-.
13. World Health Organisation. Global Action Plan for Physical Activity 2018-2020. Geneva, Switzerland; 2018.
14. Tibbitts B, Willis K, Reid T, Sebire SJ, Campbell R, Kipping RR, et al. Considerations for Individual-Level Versus Whole-School Physical Activity Interventions: Stakeholder Perspectives. **International Journal of Environmental Research and Public Health**. 2021;18(14):7628.
15. Adams J, Mytton O, White M, Monsivais P. Why Are Some Population Interventions for Diet and Obesity More Equitable and Effective Than Others? The Role of Individual Agency. **PLOS Medicine**. 2016;13(4):e1001990.
16. Atkins L, Kelly MP, Littleford C, Leng G, Michie S. Reversing the pipeline? Implementing public health evidence-based guidance in english local government. **Implementation Science**. 2017;12(1):63.
17. Swinburn BA, Sacks G, Hall KD, McPherson K, Finegood DT, Moodie ML, et al. The global obesity pandemic: shaped by global drivers and local environments. **The Lancet**. 2011;378(9793):804-14.
18. Rutter H, Savona N, Glonti K, Bibby J, Cummins S, Finegood DT, et al. The need for a complex systems model of evidence for public health. **The Lancet**. 2017;390(10112):2602-4.
19. Parkhurst JO, Abeysinghe S. What Constitutes "Good" Evidence for Public Health and Social Policy-making? From Hierarchies to Appropriateness. **Social Epistemology**. 2016;30(5-6):665-79.

For peer review only

For peer review only - http://bmjopen.bmj.com/site/about/guidelines.xhtml

20. McMahon NE. What shapes local health system actors' thinking and action on social inequalities in health? A meta-ethnography. **Social Theory & Health**. 2022.
21. Lorenc T, Petticrew M, Welch V, Tugwell P. What types of interventions generate inequalities? Evidence from systematic reviews. **Journal of Epidemiology and Community Health**. 2013;67(2):190-3.
22. Nobles JD, Radley D, Mytton OT. The Action Scales Model: A conceptual tool to identify key points for action within complex adaptive systems. **Perspectives in Public Health**. 2021:17579139211006747.
23. Swinburn BA, Kraak VI, Allender S, Atkins VJ, Baker PI, Bogard JR, et al. The Global Syndemic of Obesity, Undernutrition, and Climate Change: The Lancet Commission report. **The Lancet**. 2019;393(10173):791-846.
24. Michie S, van Stralen MM, West R. The behaviour change wheel: A new method for characterising and designing behaviour change interventions. **Implementation Science**. 2011;6(1):42.
25. Fortescue-Webb D, Evans T, Hanss K, Lamont T. Moving Matters - Interventions To Increase Physical Activity. 2019.
26. Public Health England. Whole systems approach to obesity programme: A guide to support local approaches to promoting healthy weight. London, UK: PHE Publications; 2019.
27. Bagnall A-M, Radley D, Jones R, Gately P, Nobles J, Van Dijk M, et al. Whole systems approaches to obesity and other complex public health challenges: a systematic review. **BMC Public Health**. 2019;19(1):8.
28. Rutter H, Glonti K. Towards a new model of evidence for public health. **The Lancet**. 2016;388:S7.
29. Sport England. People and places; the story of doing it differently. London, UK; 2021.
30. Karacabeyli D, Allender S, Pinkney S, Amed S. Evaluation of complex community-based childhood obesity prevention interventions. **Obesity Reviews**. 2018;19(8):1080-92.
31. Public Health England. Whole systems approach to obesity programme: Learning from co-producing and testing the guide and resources. London, UK: PHE Publications; 2019.
32. Finegood DT. Can We Build an Evidence Base on the Impact of Systems Thinking for Wicked Problems?; Comment on "What Can Policy-Makers Get Out of Systems Thinking? Policy Partners' Experiences of a Systems-Focused Research Collaboration in Preventive Health". **International Journal of Health Policy and Management**. 2020:-.
33. Bellew W, Smith BJ, Nau T, Lee K, Reece L, Bauman A. Whole of Systems Approaches to Physical Activity Policy and Practice in Australia: The ASAPa Project Overview and Initial Systems Map. **Journal of Physical Activity and Health**. 2020;17(1):68-73.
34. Kania J, Kramer M. Collective Impact. **Stanford Social Innovation Review**. 2011.
35. Nobles J, Redwood S, Jago R, Hill L, Mamluk L, Foster C. Evaluation of we can move: April 2019 – April 2021. 2021.
36. Nobles J, Wheeler J, Dunleavy-Harris K, Holmes R, Inman-Ward A, Potts A, et al. Ripple effects mapping: capturing the wider impacts of systems change efforts in public health. **BMC Med Res Methodol**. 2022;22(1):72.
37. Shearn K, Allmark P, Piercy H, Hirst J. Building Realist Program Theory for Large Complex and Messy Interventions. **International Journal of Qualitative Methods**. 2017;16(1):1609406917741796.
38. Chazdon S, Emery M, Hansen D, Higgins L, Sero R. A Field Guide to Ripple Effects Mapping. Minneapolis, USA: University of Minnesota Libraries Publishing; 2017.
39. Cooperrider D, Srivastva S. Appreciative inquiry and organisational life. Research in Organisational Change and Development. 1: JAI Press; 1987. p. 129-69.
40. Gale NK, Heath G, Cameron E, Rashid S, Redwood S. Using the framework method for the analysis of qualitative data in multi-disciplinary health research. **BMC Med Res Methodol**. 2013;13(1):117.

For peer review only

41. Sparkes AC, Smith B. Qualitative Research Methods in Sport, Exercise and Health. London, UK: Routledge; 2013.
42. Skivington K, Matthews L, Simpson SA, Craig P, Baird J, Blazeby JM, et al. A new framework for developing and evaluating complex interventions: update of Medical Research Council guidance. **BMJ**. 2021;374:n2061.
43. Moore GF, Audrey S, Barker M, Bond L, Bonell C, Hardeman W, et al. Process evaluation of complex interventions: Medical Research Council guidance. **BMJ**. 2015;350:h1258.
44. Baugh Littlejohns L, Wilson A. Strengthening complex systems for chronic disease prevention: a systematic review. **BMC Public Health**. 2019;19(1):729.
45. Stansfield J, South J, Mapplethorpe T. What are the elements of a whole system approach to community-centred public health? A qualitative study with public health leaders in England's local authority areas. **BMJ Open**. 2020;10(8):e036044.
46. Bird EL, Evans D, Gray S, Atherton E, Blackshaw J, Brannan M, et al. Opportunities to engage health system leaders in whole systems approaches to physical activity in England. **BMC Public Health**. 2022;22(1):254.
47. Bigland C, Evans D, Bolden R, Rae M. Systems leadership in practice: thematic insights from three public health case studies. **BMC Public Health**. 2020;20(1):1735.
48. Alderwick H, Hutchings A, Briggs A, Mays N. The impacts of collaboration between local health care and non-health care organizations and factors shaping how they work: a systematic review of reviews. **BMC Public Health.** 2021;21(1):753.
49. Finegood DT. The Complex Systems Science of Obesity. In: Cawley J, editor. The Oxford Handbook of the Social Science of Obesity. New York, USA: Oxford University Press, Inc.; 2011.
50. Meadows D, Wright D. Thinking in Systems: A Primer: Chelsea Green Publishing; 2008.
51. Jebb SF, D, Diez Roux A, Rutter H, Clarkson J, Frank J, Roos N, et al. Systems-based approaches in public health: Where next? UK Academy of Medical Sciences & Canadian Academy of Health Sciences; 2021.
52. Gadsby EW, Hotham S, Eida T, Lawrence C, Merritt R. Impact of a community-based pilot intervention to tackle childhood obesity: a 'whole-system approach' case study. **BMC Public Health**. 2020;20(1):1818.
53. FSG. Evaluating Complexity: Propositions for Improving Practice. 2017.
54. Walton M. Applying complexity theory: A review to inform evaluation design. **Evaluation and Program Planning**. 2014;45:119-26.
55. Hawe P, Bond L, Butler H. Knowledge theories can inform evaluation practice: What can a complexity lens add? New Directions for Evaluation. 2009(124):89-100.
56. Matheson A, Walton M, Gray R, Lindberg K, Shanthakumar M, Fyfe C, et al. Evaluating a community-based public health intervention using a complex systems approach. **Journal of Public Health**. 2017;40(3):606-13.
57. McGill E, Marks D, Er V, Penney T, Petticrew M, Egan M. Qualitative process evaluation from a complex systems perspective: A systematic review and framework for public health evaluators. **PLOS Medicine**. 2020;17(11):e1003368.
58. Orton LC, Lloyd-Williams F, Taylor-Robinson DC, Moonan M, O'Flaherty M, Capewell S. Prioritising public health: a qualitative study of decision making to reduce health inequalities. **BMC Public Health**. 2011;11(1):821.
59. Hunter D, Perkins N. Partnership Working in Public Health: The Implications for Governance of a Systems Approach. **Journal of Health Services Research & Policy**. 2012;17(2_suppl):45-52.
60. Blackman T, Harrington B, Elliott E, Greene A, Hunter DJ, Marks L, et al. Framing health inequalities for local intervention: comparative case studies. **Sociology of Health & Illness**. 2012;34(1):49-63.
61. Lynch J. Reframing inequality? The health inequalities turn as a dangerous frame shift. **Journal of Public Health**. 2017;39(4):653-60.

For peer review only - http://bmjopen.bmj.com/site/about/guidelines.xhtml

62. Crane M, Nathan N, McKay H, Lee K, Wiggers J, Bauman A. The Sustainment of Population Health Programs Requires a Systems Approach. **Health Research Policy and Systems**. 2022.
63. McGill E, Er V, Penney T, Egan M, White M, Meier P, et al. Evaluation of public health interventions from a complex systems perspective: A research methods review. **Social Science & Medicine**. 2021;272:113697.

For peer review only

For peer review only - http://bmjopen.bmj.com/site/about/guidelines.xhtml

Figure legends

Figure 1: Simple REM Example

For peer review only

Unable to Convert Image

The dimensions of this image (in pixels) are too large to be converted. For this image to convert, the total number of pixels (height x width) must be less than 40,000,000 (40 megapixels).

Figure 1

Supplement I - COREQ Checklist: Nobles et al. Implementing a Whole Systems Approach to Increase Physical Activity

Domain 1: Research team and reflexivity

Personal Characteristics

1. *Interviewer/facilitator*

James Nobles

2. *Credentials*

Ph.D.

3. *Occupation*

Research Fellow in Behavioural and Qualitative Science at the University of Bristol.

4. *Gender*

Male.

5. *Experience and training*

Seven years of qualitative research experience.

Relationship with participants

6. *Relationship established*

A relationship was present with some of the people who were interviewed / worked with for this project. James Nobles was an embedded researcher at Active Gloucestershire (the organisation who facilitate WCM), and so spent one day per week initially in their offices. During this time, he attended many meetings, workshops and events, at which he met some of the people who were interviewed. In all instances, the (prospective) interviewee knew that James' role was that of an evaluator and that he did not work for Active Gloucestershire.

7. *Participant knowledge of the Interviewer*

Where relationships were already established prior to the interview taking place, the researcher had always made clear that they were evaluating the implementation of WCM. James spoke about the different aspects of the evaluation (e.g. the research questions, the methods, the outputs etc..) at these meetings. It was important that stakeholders, some of which were future interviewees, understood why James was present in the meetings, workshops or events.

8. *Interviewer characteristics*

James had a pre-existing interest in the design, delivery and evaluation of systems approaches in Public Health. He had previously been involved in the creation of a toolkit that Local Government Authorities in England could use to design their own whole systems approach to prevent and manage overweight and obesity. The topic of physical activity was somewhat new prior to starting the evaluation of WCM in April 2019, however he – and the wider project team – were aware that

For peer review only

the previous approaches to improving physical activity had largely been ineffective in bringing about long-term, sustainable behaviour change. It is worth stating here though that the purpose of the WCM evaluation was not to determine if the population levels of physical activity changed as a result of its delivery, but rather it was mainly concerned with the learning that arises from the implementation of WCM.

Domain 2: Study design

Theoretical framework

9. *Methodological orientation and Theory* - What methodological orientation was stated to underpin the study? e.g. grounded theory, discourse analysis, ethnography, phenomenology, content analysis

This is a multi-method qualitative evaluation study.

Participant selection

10. *Sampling*

All interview participants were purposefully sampled, via one of three means:

- First, the researcher worked with members of the Active Gloucestershire team to identify people who had been involved in, or affected by, the WCM programme.
- Second, the researcher analysed the Ripple Effects Mapping outputs to identify other people or organisations who may have been involved in the periphery of WCM. Ripple Effects Mapping has been described in the main paper.
- Third, the researcher conducted a secondary analysis of a stakeholder survey that was disseminated by Active Gloucestershire amongst their contacts. The researcher looked at the responses to open-ended questions. These respondents were then sent an invitation to partake in an interview by a member of the Active Gloucestershire team.

Ripple Effects Mapping participants comprised of implementation staff (i.e. those involved in the design and delivery of We Can Move). A small group of community residents also took part in an REM workshop, as described in the paper, and all were involved in the implementation of the community-based initiative.

11. *Method of approach*

All participants were contacted via email in the first instance. They were provided with an overview of the evaluation, a description around the purpose of the interview, and then further information was included in a participant information sheet. Those who were then interested in the study were sent a consent form to complete. If participants had further questions, they were able to contact the researcher directly via phone or video-call software.

12. *Sample size*

Interview data from thirty-two people were included in this study.

13. *Non-participation* - How many people refused to participate or dropped out? Reasons?

No one refused to take part in an interview or REM session as far as we recall.

For peer review only

Setting

14. *Setting of data collection*

All interview data was collected either over the phone (i.e. telephone interviews) or via video-call software (e.g. Microsoft Teams or Zoom).

Initial REM sessions were completed via in-person workshops, and follow up workshops were all completed online using Microsoft Teams or Zoom.

15. *Presence of non-participants* - Was anyone else present besides the participants and researchers?

No

16. *Description of sample* - What are the important characteristics of the sample? e.g. demographic data, date

See paper (Page 9, Results).

Data collection

17. *Interview guide* - Were questions, prompts, guides provided by the authors? Was it pilot tested?

The interview guide is provided as a supplement to the manuscript. The interview guide was developed by the co-authors of the paper who have extensive experience in qualitative and evaluation research. The questions were designed so as to specifically probe into areas of interest. The REM outputs, as described in the paper, were also used to probe for further information in the interviews.

18. *Repeat interviews* - Were repeat interviews carried out? If yes, how many?

No

19. *Audio/visual recording* - Did the research use audio or visual recording to collect the data?

Yes – interviews were audio recorded, and REM workshops (online only) were visually and audio recorded.

20. *Field notes* - Were field notes made during and/or after the interview or focus group?

Yes.

21. *Duration* - What was the duration of the interviews or focus group?

See paper

For peer review only

22. *Data saturation* - Was data saturation discussed?

No, we planned to complete a pragmatic number of interviews per case study (n=10).

23. *Transcripts returned* - Were transcripts returned to participants for comment and/or correction?

No, although upon receiving the transcripts, authors read these concurrent to the audio recording to ensure their accuracy.

Domain 3: analysis and findings

Data analysis

24. *Number of data coders* - How many data coders coded the data?

One primary coder and a second (CF) completed a separate analysis of the data from one case study. The two authors discussed their findings and came to an agreement on the most appropriate way to present these findings.

25. *Description of the coding tree* - Did authors provide a description of the coding tree?

No

26. *Derivation of themes* - Were themes identified in advance or derived from the data?

Yes – some themes were created based upon the main areas of exploration (e.g. mechanisms of change, contextual factors, impacts etc…), and others were derived from the data where required.

27. *Software* - What software, if applicable, was used to manage the data?

NVIVO12

28. *Participant checking* - Did participants provide feedback on the findings?

We reported the findings of this work back to the implementation team and wider stakeholders to ensure that it resonated with what they had experienced. Further details are available in the manuscript (Page 8, Data analysis).

Reporting

29. *Quotations presented* - Were participant quotations presented to illustrate the themes / findings? Was each quotation identified? e.g. participant number

N/A – this is based upon reviewer comments.

30. *Data and findings consistent* - Was there consistency between the data presented and the findings?

N/A – this is based upon reviewer comments.

31. *Clarity of major themes* - Were major themes clearly presented in the findings?

N/A – this is based upon reviewer comments.

For peer review only

32. *Clarity of minor themes* - Is there a description of diverse cases or discussion of minor themes

N/A – this is based upon reviewer comments.

For peer review only

Evaluation of We Can Move

Supplement II: Interview Topic Guide

1. Can you tell me about your **role** or involvement in "we can move"?

- How did your involvement commence?
- What were your reasons for getting involved?
- If not knowledgeable of "we can move", the role with Active Gloucestershire

2. What do you **perceive "we can move" to be**?

- What do you think of when you hear "we can move"?
- What do you know about it?

3. What were your **expectations** from "we can move"?

- How did you expect it to unfold?

4. How have you seen "we can move" **evolve over time**?

- How has this happened?
- What has contributed to these adaptations to occur?

5. What have been the **main successes** of "we can move" from your perspective?

- Why did you highlight these examples?

6. To what extent do you feel that "we can move" is **focused on the right things**?

7. [Facilitators] What has **helped** "we can move" to work as it does?

- i.e. what has helped to make it successful?
- What are the important ingredients?

[Barriers] What are the **challenges**, or have been the challenges, facing "we can move"?

- What has made it more difficult?

Contextual factors that may influence the projects?

8. What **impacts have you experienced** or heard about regarding "we can move"?

- i.e. impacts on self, society, organisation, policy etc….
- Expected or unexpected?
- How did the impact occur?
- Are these as you expected?
- Did they lead to any additional impacts?

9. For you, how could "we can move" **improve** or develop in the **future**?

- What would be required to help achieve this?

10. What do you **perceive your role to be** in "we can move" in the future?

For peer review only

BMJ Open

Navigating the river(s) of systems change: a multi-methods, qualitative evaluation exploring the implementation of a systems approach to physical activity in Gloucestershire, England

Journal:	*BMJ Open*
Manuscript ID	bmjopen-2022-063638.R1
Article Type:	Original research
Date Submitted by the Author:	23-Jun-2022
Complete List of Authors:	Nobles, James; University of Bristol, Population Health Sciences; NIHR ARC West<br>Fox, Charlotte; Royal Borough of Windsor and Maidenhead Council<br>Inman-Ward, Alan; Active Gloucestershire<br>Beasley, Tom; Active Gloucestershire<br>Redwood, Sabi; Bristol, Population Health Sciences; NIHR ARC West<br>Jago, Russ; University of Bristol, Centre for Exercise, Nutrition and Health Sciences; NIHR ARC West<br>Foster, Charlie ; University of Bristol Faculty of Health Sciences
**Primary Subject Heading**:	Public health
Secondary Subject Heading:	Qualitative research, Research methods, Health policy
Keywords:	PUBLIC HEALTH, Health policy < HEALTH SERVICES ADMINISTRATION & MANAGEMENT, Organisation of health services < HEALTH SERVICES ADMINISTRATION & MANAGEMENT, QUALITATIVE RESEARCH, SOCIAL MEDICINE

SCHOLARONE™
Manuscripts

I, the Submitting Author has the right to grant and does grant on behalf of all authors of the Work (as defined in the below author licence), an exclusive licence and/or a non-exclusive licence for contributions from authors who are: i) UK Crown employees; ii) where BMJ has agreed a CC-BY licence shall apply, and/or iii) in accordance with the terms applicable for US Federal Government officers or employees acting as part of their official duties; on a worldwide, perpetual, irrevocable, royalty-free basis to BMJ Publishing Group Ltd ("BMJ") its licensees and where the relevant Journal is co-owned by BMJ to the co-owners of the Journal, to publish the Work in this journal and any other BMJ products and to exploit all rights, as set out in our licence.

The Submitting Author accepts and understands that any supply made under these terms is made by BMJ to the Submitting Author unless you are acting as an employee on behalf of your employer or a postgraduate student of an affiliated institution which is paying any applicable article publishing charge ("APC") for Open Access articles. Where the Submitting Author wishes to make the Work available on an Open Access basis (and intends to pay the relevant APC), the terms of reuse of such Open Access shall be governed by a Creative Commons licence – details of these licences and which Creative Commons licence will apply to this Work are set out in our licence referred to above.

Other than as permitted in any relevant BMJ Author's Self Archiving Policies, I confirm this Work has not been accepted for publication elsewhere, is not being considered for publication elsewhere and does not duplicate material already published. I confirm all authors consent to publication of this Work and authorise the granting of this licence.

For peer review only

Title

Navigating the river(s) of systems change: a multi-methods, qualitative evaluation exploring the implementation of a systems approach to physical activity in Gloucestershire, England

Authors and institutions

James Nobles[1,2 *], Charlotte Fox[3], Alan Inman-Ward[4], Tom Beasley[4], Sabi Redwood[1,2], Russell Jago[1,2,5], and Charlie Foster[5]

[1] The National Institute for Health Research Applied Research Collaboration West (NIHR ARC West) at University Hospitals Bristol National Health Service Foundation Trust, United Kingdom

[2] Population Health Sciences, Bristol Medical School, University of Bristol, United Kingdom

[3] Royal Borough of Windsor and Maidenhead Council, United Kingdom

[4] Active Gloucestershire, Gloucester, United Kingdom

[5] Centre for Exercise, Nutrition and Health Sciences, School for Policy Studies, University of Bristol, United Kingdom

*Corresponding author: James Nobles, University of Bristol, james.nobles@bristol.ac.uk

For peer review only

Structured abstract

Objectives: Systems approaches aim to change the environments in which people live, through cross-sectoral working, by harnessing the complexity of the problem. This paper sought to identify: a) the strategies which support the implementation of We Can Move (WCM), b) the barriers to implementation, c) key contextual factors that influence implementation, and d) impacts associated with WCM.

Design: A multi-methods evaluation of WCM was completed between April 2019 and April 2021. Ripple Effects Mapping (REM) and semi-structured interviewers were used. Framework and content analysis were systematically applied to the dataset.

Setting: We Can Move - a physical activity orientated systems approach being implemented in Gloucestershire, England.

Participants: 31 stakeholder interviews and 25 stakeholders involved in 15 REM workshops.

For peer review only - http://bmjopen.bmj.com/site/about/guidelines.xhtml

Results: A white-water rafting analogy was developed to present the main findings. The successful implementation of WCM required a facilitative, well-connected, and knowledgeable guide (i.e. the lead organisation), a crew (i.e. wider stakeholders) who's vision and agenda aligned with WCM's purpose, and a flexible delivery approach that could respond to ever-changing nature of the river (i.e. local and national circumstances). The context surrounding WCM further strengthened and hampered its implementation. Barriers included evaluative difficulties, a difference in stakeholder and organisational perspectives, misaligned expectations and understandings of WCM, and COVID-19 implications (COVID-19 also presented as a facilitative factor). WCM was said to strengthen cohesion and collaboration between partners, benefit other agendas and policies (e.g. mental health, town planning, inequality), and improve physical activity opportunities and environments.

Conclusions: This paper is one of the first to evaluate a systems approach to increasing physical activity. We highlight key strategies and contextual factors that influenced the implementation of WCM and demonstrate some of the wider benefits from such approaches. Further research and methodologies are required to build the evidence base surrounding systems approaches in public health.

Key words

Systems approach; systems science; complex adaptive systems; public health; participatory research; ripple effects mapping; multi-method; implementation.

Strengths and limitations of this study

- A multi-methods, innovative approach was used to comprehensively answer the evaluative aims of this research.
- Ripple Effects Mapping was used to evidence wider impacts of WCM, but also to inform the interviewee sampling and questioning.
- The data collection approach was predominantly completed virtually due to COVID-19 restrictions which lessened the utility of REM.

Word count: 8678

For peer review only

Main text

1. Introduction

Global data suggest that a quarter of adults and three quarters of adolescents are not engaged in recommended amounts of physical activity (1, 2). National data in England paint a similar picture. Physical activity behaviour is socially patterned, and people who live in more deprived areas and who are from ethnically diverse groups have lower levels of physical activity (3). These low physical activity levels can contribute towards poor health and wellbeing outcomes (4-6), as well as having notable direct and indirect health and social costs (4, 7).

The World Health Organisation recently explored the complex drivers of low physical activity (8). They argued that the population levels of physical activity are the outcome of an interconnected, interdependent, and evolving web of causal factors – in other words, a complex adaptive system. They outlined 45 causal factors that spanned multiple themes; from individual factors (e.g. motivation, self-efficacy, family support), to socio-political factors (e.g. employment status, socio-economic status, disposable income) to transport and environmental factors (e.g. public transport, air quality, walking infrastructure) (8). Others, including a 2021 commission by the Lancet, corroborate these arguments (6, 9-13).

Historically, interventions to increase physical activity have largely focused on individual agency and decision-making as the primary mechanism of change (24). These interventions seek to change individual behaviours, either through short-term educational approaches or through the provision of physical activity opportunities (14). These approaches are also politically appealing because they require minimal political will to implement, they are relatively low cost, they are easy to measure, and there is an evidence base that can be used to justify their delivery (15-20). However, individual level interventions can exacerbate, rather than reduce, health inequalities, and seldom change the environments that people move and function within (15, 17, 21).

Approaches that require lower levels of individual agency have the potential for broader reach, and more importantly, reducing inequalities (15). A systems approach aims to influence multiple parts of the system concurrently by drawing on the expertise and resources of individuals, communities, organisations, and sectors (6, 22, 23). Systems approaches have the potential to reshape the environments that people interact with, and in doing so, can change default and automatic behaviours (11, 15, 22, 24, 25). These approaches can adapt over time in response to dynamic contexts, the engagement from stakeholders, and the observed impacts (intended or unintended) across the system (6). Therefore, no two systems approaches will look, function, or feel the same. There is broad

For peer review only - http://bmjopen.bmj.com/site/about/guidelines.xhtml

consensus that systems approaches are required to help address many of the complex public health issues being faced (8, 11, 18, 23, 26-29).

Practice has now outstripped the research and evidence base. During the last 10 years, there has been a groundswell in the implementation of systems approaches (27, 30). For example, Sport England – a national non-departmental government organisation – has allocated over £100 million for Local Delivery Pilots. The purpose of the Local Delivery Pilots are to test out novel, systemic, and joined up approaches that have the long-term ambition of improving population physical activity, and health, outcomes (29). The Office for Health Improvement and Disparities (formerly Public Health England) has also invested substantial resource to support local government authorities to implement a whole systems approach to obesity (26, 31). Similar patterns and investments have been observed internationally (27, 30). Despite the growing interest in, and implementation of, systems approaches in Public Health, there is a distinct lack of published research surrounding their evaluation (18, 27, 30, 32, 33). In this paper, we will provide the results from one such evaluation.

We Can Move (WCM) began its implementation in April 2018. Through a systems approach, it aimed to influence the physical activity levels across the Gloucestershire population, with a specific focus on populations with very low levels of activity (less than 30 minutes per week). Active Gloucestershire is a voluntary sector organisation that co-ordinated WCM, otherwise known as the 'backbone organisation' (34). Together, with a small number of stakeholders from other organisations, they made up the WCM implementation team. The implementation team worked with a wide variety of community groups, organisations and system leaders from multiple sectors to overcome the barriers facing individuals (e.g. opportunities for physical activity), communities (e.g. the quality of our physical and social environments), and the wider system (e.g. the policies and people that make the system work as it does). It was anticipated that WCM would not only benefit the physical activity levels in Gloucestershire, but many other associated agendas such as climate change, overweight and obesity, and mental health. Box 1 provides further details about WCM.

In April 2019, Active Gloucestershire commissioned the National Institute for Health Research Applied Research Collaboration West (NIHR ARC West) to evaluate WCM. This paper will: a) identify the strategies which supported the implementation of WCM , b) highlight the barriers to WCM being implemented, c) illustrate key contextual factors that influenced its implementation (i.e. factors that are not part of the intervention per se, or were already established pre-implementation (35, 36)), and d) demonstrate the range of impacts associated with WCM being implemented. The WCM programme continued beyond the evaluation period.

For peer review only

Box 1: Description of We Can Move

About WCM: In 2017, Active Gloucestershire secured funding to research alternative approaches to improving the population levels of physical activity. During this time, they developed a high-level *Theory of Change* for a new approach, initially named "Gloucestershire Moves". The Theory of Change included systems science, behaviour change principles, and social movement building (for further information, see Nobles *et al.* (37)). It would see methods like systems mapping being used to understand the complexity of the problems being faced locally, and then models such as the Behaviour Change Wheel (24) helping to inform the development of interventions.

During 2020, the Gloucestershire Moves changed its name to "We Can Move".

The vision for WCM was to inspire, connect, and enable individuals, communities and organisations to help the least active, move more.

It hoped to achieve this by engaging a wide variety of community groups, organisations and system leaders to remove the barriers facing individuals (e.g. the opportunities for physical activity), communities (e.g. the quality of our physical and social environments), and the wider system (e.g. the policies and people that make the system work as it does).

The Theory of Change was initially tested on the Falls Prevention Initiative and the Community-Based Initiative (the two case studies being evaluated). The Theory evolved slightly throughout this piloting, before being phased into all of Active Gloucestershire's work between 2018 and 2021.

WCM cost an estimated £3,000,000 to implement between April 2018 and April 2021.

Further information is available in Nobles *et al.* (2021).

About Active Gloucestershire: Active Gloucestershire are a small voluntary sector organisation funded through national and local grants, local public sector organisations, and charitable donations. Within WCM, Active Gloucestershire's role was to facilitate this approach – also known as the "backbone organisation". Prior to WCM, Active Gloucestershire's role was to deliver physical activity sessions and other types of interventions to the public. This often meant working with those who were already physically active.

Setting: WCM was implemented in Gloucestershire, England. Population of approx. 640,000 people. Demographic is aging, 95% white British, and has areas of high deprivation. An estimated 130,000 adults do less than 30 minutes physical activity per week, and 52.7% of children do less than six hours of activity per week.

2. Methods

2.1 Study design

This study draws on the data from a multi-methods evaluation of the WCM programme. Further details of the whole evaluation are available elsewhere (37). Here, we will report on the findings from a set of stakeholder interviews and Ripple Effects Mapping (REM) workshops. In brief, REM is a participatory, qualitative method for understanding and visualising the wider intended and unintended impacts of a project or programme (described in depth later and elsewhere (38)). The data pertaining to these two methods were drawn upon here as they provide in depth insights into how WCM functions, the contextual factors which influence its implementation, and to highlight an array of impacts associated with WCM. These two qualitative methods are complimentary, and the findings can be triangulated to comprehensively address the research questions.

It is important to note here that the lead researcher (JN) was embedded in the Active Gloucestershire organisation for one day per week. As part of this role, they attended programme-related meetings and workshops, met with wider stakeholders, and consequently, created meaningful relationships with the implementation team. The embedded nature of this role enabled the researcher to understand the nuance of the WCM programme, and subsequently, was seen to bolster the evaluation effort (39).

For peer review only - http://bmjopen.bmj.com/site/about/guidelines.xhtml

Further details related to the methodology is available in Online Supplement I (COREQ Checklist).

2.2 Case studies

Three aspects of WCM were identified to create case studies which would help to explore how the Theory of Change (see **Error! Reference source not found.**) was implemented in practice, and moreover, how each case study contributed towards the WCM programme. These case studies provided artificial and pragmatic boundaries around a complex, messy programme that would otherwise be unwieldy to evaluate within the resource constraints.

Two case studies focused on smaller projects situated within WCM (Falls Prevention Initiative and a Community-based Initiative) and the last explored, more broadly, how WCM engaged with organisations and wider stakeholders around physical activity (Table 1). Each case study was identified and agreed upon through discussions between the research team and Active Gloucestershire. An adaptive evaluation framework was applied to each case, meaning that different methods could be applied as needed to best answer the evaluation questions. At the same time, some methods, such as semi-structured stakeholder interviews and REM, were applied across all studies to ensure consistency in the type of data collected and insights generated. This further strengthened the rationale to centre this paper on the data and results related to interviews and REM.

Table 1: Case descriptions

Case 1: Falls Prevention Initiative	Case 2: Community-based Initiative	Case 3: WCM movement building
**Background**: Gloucestershire has an aging population. Older adults are at a greater risk of falling and being admitted to hospital due to falling.<br><br>**About**: A healthcare organisation funded Active Gloucestershire to develop a falls prevention initiative for the county, with the stated outcome of reducing falls admissions to the Accident and Emergency department.<br><br>Active Gloucestershire worked with community members and professionals to design and develop Fall-Proof. Behaviour change techniques were used to design a behaviourally informed intervention.<br><br>Fall-Proof aimed to increase older adults' awareness of falls risk, to encourage strength and balance exercises at home, and to increase referrals to community-based strength and balance classes.	**Background**: Barton and Tredworth is a small, culturally diverse, deprived area in Gloucester.<br><br>**About**: Active Gloucestershire wanted to start working in a hyper-local manner that responds to, and works with, residents.<br><br>As part of WCM, Active Gloucestershire received funding from a national organisation to work with the local community, including a group of local women, in Barton and Tredworth. These women wanted to create opportunities for other women, particularly Muslim women, to participate in physical activity.<br><br>This group organised many opportunities for physical activity, linked in with local organisations, and championed physical activity in their communities. Active Gloucestershire facilitated the group.	**Background**: Social movement building is a core component of the WCM Theory of Change. It provides a framework for stakeholders and organisations to integrate into, and take ownership of, WCM.<br><br>**About**: WCM seeks to work with multiple organisations and sectors to make Gloucestershire a more conducive place for physical activity. WCM encourages stakeholders to see their place in the system, to see how they could contribute to making physical activity the norm, and to see that many different agendas would benefit from improved collective working.<br><br>Active Gloucestershire supported and facilitated WCM's implementation, alongside providing opportunities for organisations to engage in and support WCM, and engage with each other.

For peer review only - http://bmjopen.bmj.com/site/about/guidelines.xhtml

2.3 Data collection

We focus here on the data gathered using semi-structured interviews and REM. We planned to recruit 10 stakeholders per case study to participate in a semi-structured interview (*n*=30 total). This sample size was informed by the resource available and the need to reflect a balance of views across the case studies. Prospective interviewees were purposefully sampled to gather a diverse range of perspectives via three main channels: a) through conversations with the WCM implementation team (i.e. those involved in the design and delivery of WCM, *predominantly* Active Gloucestershire staff); b) using the REM outputs; and c) using data gleaned from an annual stakeholder survey. The survey was developed by the evaluation team to understand the levels of engagement and involvement of wider stakeholders in WCM. Thirty-seven stakeholders completed this survey in 2020 and 2021. We purposefully sampled interview participants based on their responses to elicit maximum variation in the data set. In summary, interviewees were either involved in, or affected by, the implementation of WCM (or the specific case study within WCM).

A topic guide (Online Supplement II) was used to provide consistency within the stakeholder interviews. It paid attention to how stakeholders were involved in WCM, key contextual factors, the strategies facilitating and the barriers inhibiting its implementation, and the perceived impacts associated with WCM. Probing questions were then tailored to each interviewee based on content discussed and the REM output which they may be associated with (further information in Table 2). For example, an interviewee may have been asked how their work contributed towards an impact that had been recorded in an REM output. Interviews were conducted by the same researcher (JN) using online videoconferencing platforms. Interviews typically lasted 30 – 60 minutes.

Regarding REM, we adapted the original in-depth REM technique from Chazdon *et al.* (40) so that it captures the temporal aspects of systems change efforts. The adaptations to this method have been reported elsewhere (38). In brief, data were gathered via participatory workshops with the implementation team, and were led by an experienced facilitator (JN). The facilitator guided participants through a series of activities (Table 2) that created a visual REM output which documented the actions and activities undertaken, along a timeline, and their associated initial and wider impacts.

The first REM workshops were conducted face-to-face with the whole WCM implementation team, lasting 120-150 minutes. The implementation team split into smaller groups to focus on particular aspects of, or a project within, WCM. For example, one group focused on the Falls Prevention Initiative

For peer review only - http://bmjopen.bmj.com/site/about/guidelines.xhtml

and another on the Community-based Initiative. There was time within the workshops for participants to create multiple REM outputs. Due to COVID-19 restrictions, all follow up workshops were carried out using online videoconferencing platforms with smaller groups (based on the groups they worked during the first in-person REM workshop). Follow-up workshops (60-90 minutes) aimed to update previous REM outputs with new actions, activities, and impacts that occurred throughout the last 3-4 months. Data were recorded in a paper-based format in the face-to-face sessions, and data from subsequent follow-up workshops were entered into Vensim PLE, and with permission, conversations were recorded directly through online videoconferencing platforms. Further details on how we applied REM in the WCM evaluation is available in a methodological paper (38). See Figure 1 for a simplified and illustrative REM output.

Data from the two methods were collected between May 2019 and March 2021. The REM workshops were generally conducted prior to the participant interviews so that the outputs could inform the interview questions. We completed the interviews first for the Community-based Initiative by May 2020, (first and last REM session: May 2019 and July 2020), then the Falls Prevention Initiative by October 2020 (first and last REM session: December 2019 and March 2021), and finally, for the WCM movement building case study by January 2021 (first and last REM session: December 2019 and March 2021). Table 2: Summary of REM stages (38)

REM Stages	Description
1. Introduction to REM	The facilitator gave a brief presentation to introduce the REM method. This included background to the method, the limitations of traditional evaluation methods, and an outline of the approach used in the REM workshops. Not required in follow-up workshops.<br>*Time allowed*: 10-15 minutes
2. Peer/team-based discussions	Participants split into pairs or small project groups and had conversations about the work they are involved in, what they are proud of, what has gone well within their work, and what has been challenging. These discussions were guided by principles from Appreciative Inquiry (38, 41). Conversations served as a warm-up to the main activity.<br>*Time allowed*: 10-20 minutes
3. Mapping the impacts	Each project group was provided with a large piece of paper that had a timeline placed longitudinally across it. The timeline spanned from the beginning of WCM (April 2018) to the present time (December 2019). Participants were asked to think about the main activities or actions that were carried out throughout this timeframe, and to write these onto the map at the time which they occurred. They were then asked to map out the impacts associated with the respective activities. Arrows were draw between activities and impacts to denote the relationship. Participants were encouraged then to map any additional "ripples" that manifest following this activity and / or impact. The facilitator assisted groups where needed. During this time, groups were able to work on REM outputs associated with two to three projects / areas of work.<br>*Time allowed*: 60-90 minutes.
4. Reflecting on the impacts	Part way through the previous stage, the facilitator asked participants to consider additional questions when mapping. These included: a) identifying intended and unintended impacts, b) stating which organisations / stakeholders / groups were involved or affected, c) financial implications, d) what else may have contributed to impacts occurring, and e) how these activities / impacts link with wider WCM work. Recurring trends may also have been apparent in their outputs. When organisations / stakeholders / groups were identified, these provided the evaluation team with potential interview participants.<br>*Time allowed*: Concurrent to stage 3.

For peer review only - http://bmjopen.bmj.com/site/about/guidelines.xhtml

5.	Most and least significant changes	Once most of the actions, activities and impacts are mapped out, the facilitator then asked the groups to identify what they considered to be the most- and least-significant changes in their output. Groups had time to discuss what may have contributed to these changes occurring as they did, and whether in retrospect, they would do anything differently. Data were not recorded for evaluative purposes at this stage.<br>*Time allowed*: 10 minutes
6.	Group feedback and learning	The final part of the workshop provided time for participants to feedback to the group the main aspects of their work that was mapped, what their reflections on the map were, and what they had learnt from the REM process. The facilitator guided this discussion.<br>*Time allowed*: Remaining time.

2.4 Data analysis

Interview recordings were transcribed verbatim and uploaded into NVivo 12 for data management. A framework analysis approach was applied to all qualitative data within the WCM data set (42), which included the data from interviews and the REM outputs. The initial framework was devised around core features of interest, including contextual factors, implementation strategies, and intended and unintended outcomes. Data pertaining to the three case studies were analysed independently, so that similarities and differences could be drawn out. Additional content analyses (43) were used to quantify certain aspects of the REM outputs (e.g. number of organisations involved, number of policies influenced etc…). More information regarding the analysis of REM outputs is available in a methodological paper (38). All data were analysed by the lead researcher (JN) and supported by a second researcher (CFox) to minimise bias. To increase the dependability of the findings, a researcher (JN) had regular discussions with the implementation team and the wider stakeholders to ensure preliminary findings resonated with their experiences. Findings, not data, were triangulated from the interviews and REM as part of the framework analysis for the purpose of this paper. The majority of the findings related to a) reasons for involvement, b) implementation strategies, c) barriers to implementation, and d) contextual factors originate from the analysis of the interviews, bolstered by the REM analysis. The findings regarding the impacts and outcomes predominantly arise from the REM analysis and strengthened by the interview findings.

[INSERT FIGURE 1 HERE]

2.5 Patient and public involvement

No patient and public involvement.

3. Results

For peer review only

Thirty-one interviews were conducted across the three case studies. Interviewees represented local government authorities, health care services and commissioning, voluntary and community sector organisations, local elected members, community residents, and Active Gloucestershire staff. Fifteen REM outputs were created with the input of 25 participants (n=17 implementation staff, n=8 community residents involved in the Community-based Initiative). Seven outputs were updated over the course of a year (further information available in Nobles *et al.* (38)). One output related to the Falls Prevention Initiative and Community-based Initiative respectively, and the remaining outputs were included within the third case study (WCM movement building). The final themes from the analysis of the interview and REM outputs are presented under five key headings: 1) reasons for stakeholder involvement; 2) implementation strategies; 3) barriers to implementation; 4) contextual factors; and 5) impacts and outcomes (Table 3). Text in **bold** below represents a sub-theme.

We have developed a white-water rafting analogy to help explain, and interconnect, many of the findings below. The analogy illustrates how an approach like WCM (represented by the raft) requires a guide (i.e. Active Gloucestershire), a crew (i.e. wider stakeholders), and the ability to negotiate the dynamic nature of the river (i.e. the broader context and circumstances which influence implementation).

For peer review only

For peer review only - http://bmjopen.bmj.com/site/about/guidelines.xhtml

Table 3: Framework analysis

	Case 1: Falls Prevention Initiative[a]	Case 2: Community-based Initiative[b]	Case 3: WCM movement building[c]
**1. Reasons for involvement**	• Project aligned with, and could help, organisational priorities.<br>• Belief in the project and the associated benefits.<br>• Opportunity to network and share learning across organisations.<br>• Perceived as a "quick win".	• Experienced, or belief in, benefits of physical activity.<br>• Want to increase physical activity opportunities and levels.<br>• Believe in the wider benefits to society and the community (i.e. "not just about physical activity").<br>• Opportunity to learn about new approaches.<br>• Live in the local area.	• Shared priorities, passions and values.<br>• Personal, organisational or sectoral benefits from engagement.<br>• Contractual obligations.
**2. Implementation strategy**	• Backbone role:<br>○ Links with community.<br>○ Signposting to materials.<br>○ Marketing expertise.<br>○ Bringing people together.<br>• Key stakeholders involved in design of the initiative.<br>• Capitalise on pre-existing relationships.<br>• Dissemination via influential stakeholders and residents.<br>• Formal conduits between organisations.<br>• Behaviour Change Wheel approach to intervention design.<br>• COVID-19 causing statutory organisations to consider alternative ways of working.	• Backbone role:<br>○ Pivotal mechanism.<br>○ Facilitative.<br>○ Provide knowledge, expertise, and credibility.<br>○ "A way of working".<br>• Steering group role:<br>○ Understand local needs and wants.<br>○ Tailored, non-judgemental meetings.<br>○ Proactive and relatable community members.<br>○ Central hub to nurture ideas.<br>○ Disseminate information to community about physical activity and related opportunities.<br>○ Champion physical activity for community.<br>• Engage the right people and organisations.<br>• Create a collective and shared vision.<br>• Establish effective communication channels between stakeholders.<br>• Understand root causes of low physical activity, driven by data and insight.<br>• "Go with the flow".<br>• Keep it local & draw on community assets.<br>• Selling physical activity through persuasive and creative communications.	• Backbone role:<br>○ Facilitating (and disrupting) the system.<br>○ "Joining the dots" - fostering new relationships.<br>○ Linking in with, and establishing, professional networks.<br>○ Influencing the system and system leaders.<br>○ Imparting knowledge and expertise.<br>○ Designing and delivering projects.<br>○ Supporting other organisations.<br>○ Fiducial responsibilities.<br>○ Champion WCM and physical activity.<br>• Adaptive and strong governance for WCM.<br>• Developing strong foundations for WCM:<br>○ Passion of Active Gloucestershire team.<br>○ Informal meetings.<br>○ Council and senior leadership support.<br>○ Alignment with strategic priorities in the county.<br>○ Funding in place.<br>• Working with the right people who have a shared vision, agenda and purpose.<br>• Focus on building strong relationships between people and organisations.<br>• Events and networks can initiate and catalyse relationship building.<br>• Develop conduits between organisations and WCM.<br>• Ability of WCM to "read the river" – timing and conditions.<br>• Celebrating success.<br>• COVID-19 led to new opportunities, funding, and partnerships.
**3. Barriers to implementation**	• Clarity on roles, responsibilities, and involvement of wider stakeholders.<br>• Communication between backbone organisation and wider stakeholders.	• Overcoming bureaucracy, red tape, and administrative challenges.<br>• Monitoring "success" of Community-based Initiatives.	• Stakeholder understanding, communications, and perceptions of WCM.<br>• Clashing of mindsets.<br>• (Continued) Engagement in a social movement.

For peer review only

	• "Demonstrating impact" - evaluation, measurement, and monitoring of complex projects.<br>• Managing conflicting stakeholder tensions, mindsets, and ways of working.<br>• COVID-19 impact on delivery and participation.<br>• Linking the Falls Prevention Initiative to WCM.<br>• Backbone organisation capacity.<br>• Length of time to design intervention.	• At odds with transactional mindsets of organisations and senior leadership.<br>• Limited stakeholder engagement.<br>• Acknowledge challenges within a steering group (format, aims, opposing views).	• Inconsistent preferences surrounding evaluation.<br>• COVID-19 prevented progress and collaboration across WCM.<br>• COVID-19 impact on stakeholder engagement, and prioritisation of WCM by organisations.
**4. Contextual factors**	*Facilitative contextual factors*<br>• History of local falls prevention work.<br>• Organisational strengths of Active Gloucestershire.<br>*Inhibitory contextual factors*<br>• Institutional transactional ways of working.<br>• Active Gloucestershire going through transition period.	*Facilitative contextual factors*<br>• Active Gloucestershire as a trusted, well-connected, and established organisation.<br>• Established Voluntary and Community Sector and history of community building.<br>• Mindset shift occurring in some organisations.<br>*Inhibitory contextual factors*<br>• Negative perception towards previous approaches - "[locality was] a picked-on area".<br>• Varied demographic of local community.<br>• Cultural norms against physical activity.	*Facilitative contextual factors*<br>• Active Gloucestershire well-connected, respected, and knowledgeable organisation.<br>• "WCM pushing on an open door".<br>*Inhibitory contextual factors*<br>• Demographic profile of Gloucestershire.<br>*Both facilitative and inhibitory*<br>• COVID-19 implications.
**5. Impacts and outcomes**	• Awareness, use and uptake of the Falls Prevention Initiative work.<br>• Quality of the Falls Prevention Initiative materials.<br>• Stakeholders feeling part of something bigger.<br>• Wider engagement in Falls Prevention Initiative materials (locally and nationally).<br>• Limited impact on referrals to strength and balance providers.	• Perceived increases in community physical activity.<br>• Improved / changed physical activity infrastructure and provision.<br>• Enhanced community engagement, cohesion, and empowerment.<br>• Growth, reach and self-organisation of work.<br>• Influence on, and buy-in from, wider sectors.<br>• New money and funds generated.<br>• Steering group:<br>○ Group ownership changed over time.<br>○ Buy-in from local councillors.<br>○ Feel part of something bigger.<br>○ Feel empowered and accomplished.	• Successful buy-in and support for WCM.<br>• Mindset shifts within organisations and senior leaders.<br>• "Linking up the system" – new relationships created.<br>• "Systems change happening in pockets".<br>• Beneficial impacts and influence on communities, organisations and sectors.<br>• Uptake of physical activity and interventions.<br>• New money and funds generated.<br>• New projects established.<br>• Other areas adopting / interested in WCM.<br>• Supporting, benefiting and aligning with other strategic agendas.

[a] **Falls Prevention Initiative data collection**: 12 stakeholder interviews, one REM output (created over four iterations).

[b] **Community-based Initiative data collection**: 10 stakeholder interviews, one REM output (created over five iterations).

[c] **WCM movement building data collection**: Nine stakeholder interviews, 13 REM outputs (five created over multiple iterations).

For peer review only

For peer review only - http://bmjopen.bmj.com/site/about/guidelines.xhtml

3.1 Reasons for involvement

Stakeholders frequently had multiple reasons for getting involved in WCM. Across the three cases, stakeholders believed in the **co-benefits** associated with WCM and physical activity (e.g. mental health, social connections, health inequalities), which could be realised by individuals, communities, organisations, and wider sectors (e.g. Transport or Social Care). Many also noted that WCM aligned with the **strategic priorities of their own organisation**, and so their involvement in WCM would be mutually beneficial. For example, one stakeholder highlighted how WCM could benefit a council agenda around adverse childhood experiences (ACEs):

> *"ACEs is one of the sort of Health and Wellbeing Board priorities and it has been a priority since 2018. Part of that and the research around ACEs was around, we know that you can build resilience through regular sports participation and we're using the five ways to wellbeing as our sort of resilience building tool." – Participant 2, Case 3, County council public health*

Other reasons were more specific to individual cases. Some bought into the Falls Prevention Initiative because they believed it would bring about "quick-wins" – most notably, reductions in the number of people being admitted to hospital because of falling. On the other hand, in the Community-based Initiative, several community residents became involved because they lived locally and wanted to benefit the community, whilst others wanted to learn about the WCM Theory of Change (**Error! Reference source not found.**).

3.2 Implementation strategies

Our white-water rafting analogy is particularly useful here to present the three main themes. These include: 1) the role of the guide (i.e. the backbone organisation), 2) developing the right crew (i.e. wider stakeholders), and 3) the ability of WCM to "read the river" (i.e. local context and circumstances). These factors are interconnected and interdependent, as explained below, using the white-water rafting analogy.

A fundamental **role of a guide** is to steer the raft by talking to, motivating, and inspiring the crew. In WCM, Active Gloucestershire held the role of the guide, also referred to as the "backbone organisation". Whilst the backbone role varied across the three case studies, its importance was widely acknowledged by participants. For example, in the Community-based Initiative (Case 2), the backbone role was held by one staff member of Active Gloucestershire. Interviewees stated that they were pivotal in that they facilitated relationships between stakeholders, that they provided knowledge and expertise (about physical activity and about local networks of stakeholders and organisations), and that their role – and the way in which they worked – was "*more than a job*" (Participant 6, Case

For peer review only

1, Community member). In the context of WCM (Case 3), the backbone role was held by Active Gloucestershire as an organisation. Active Gloucestershire were seen to be centrally involved in facilitating how the physical activity system was organised – from the stakeholder networks to the way in which funding was distributed within the sector. This was referred to as a "*behind the scenes*" role (Participant 7, Case 3, Local third sector organisation). Thus, they were believed to have substantial influence given their strong connections to senior leaders across Gloucestershire, further reinforced by their credibility as an organisation. Interviewees also spoke of the intricate knowledge (of people, places, and communities) and extensive expertise (in physical activity, project management, and behaviour change methodology) that Active Gloucestershire held and were able to share with others. A large part of the backbone role was to "*join the dots*" (multiple interviewees references) – to bring people, organisations, and their agendas, together. They did this via formal (e.g. setting up stakeholder networks) and informal (e.g. email introductions) methods. Several interviewees reported that Active Gloucestershire provided in-depth advisory support to their organisation given that they were relatively new to the county and/or work in the Public Health sphere. At their core, Active Gloucestershire were passionately championing, facilitating and co-ordinating WCM.

> *I think that what Active Glos did getting all those people together, was the contacts that they've got and the networks that they've got. They're very different from our health network, so I think that was brilliant. It did make sure that the community voice from a lot of different perspectives remained through those [Falls Prevention Initiative] planning stages definitely. – Participant 3, Case 1, NHS commissioning*

> *I think it's imperative to have somebody on the ground that knows the local area, can build a relationship with local people, they've got that local knowledge, they can connect like-minded people and just offer that bit of support – Participant 3, Case 2, City council*

To negotiate the turbulence of the river, a raft needs more than a guide, it needs a crew. This is where the implementation and success of WCM was largely hinged on its engagement with wider stakeholders. Interviewees said that Active Gloucestershire placed a lot of effort on **developing the "right" crew**; the people and organisations to work with as part of WCM. The "right" people and organisations typically comprised those whose *vision* (i.e. how they work and what they are aspiring to) aligned with that of WCM, and whose *agenda* (i.e. the things that are important /prioritised within their organisation) would benefit from the work of WCM. These two elements, vision and agenda, enabled wider stakeholders to see value in WCM and to better see their role in WCM. Furthermore, interviewees spoke about the personality and behavioural characteristics (or "mindset" – i.e. way of thinking about problems and actions) of wider stakeholders who were influential within WCM. This

For peer review only - http://bmjopen.bmj.com/site/about/guidelines.xhtml

included an ability to build strong relationships, to look and work beyond their own organisational priorities (and thus acknowledge that they work within a system rather than silos), and to feel comfortable working – or attempting to work – in a different, and perhaps more collaborative and equal, manner. These crew members often acted as conduits for information about WCM to flow through to their senior management, therefore working as a strategy to increase organisational buy-in to WCM.

> *I have always been that new way, I've always done this sort of way, I always think connecting the dots is much better, more effective so I've never worked in that sort of old way and that silo approach I guess. So I think there might be a bit of challenge around how they work and you know bringing people – great, get everyone in a room, get everyone interested but then you might get some people that would just automatically flip back to, right what's on priorities, what's on the agenda I've got to do today. – Participant 2, Case 3, County council public health*

To identify the "right" people, Active Gloucestershire staff invested substantial time in attending and organising networks (via REM analysis, at least 23 networks were attended or organised). These networks often led to serendipitous outcomes; for example, Active Gloucestershire staff met new people and organisations, organised meetings outside of the networks, which subsequently led to unplanned joint working that would benefit the overall implementation of WCM (and the work of the other organisation). We noted, through the REM analysis, that 82 organisations had been engaged in WCM throughout the data collection (April 2019-January 2021), and there were many examples of these unanticipated partnerships in the dataset. Most of the work, and relationships developed, in the Community-based Initiative (Case 2) came about because of an initial network meeting held by Active Gloucestershire called "Joining the Dots" which was attended by approximately 65 local organisations. However, the REM and interview analyses also suggest that time was needed to develop meaningful, trusting relationships, while the length of time required varied dependent on the stakeholder or organisations engaged with.

With the guide and crew established, the final key strategy was the ability for WCM to "**read the river**". Interviewees described how WCM (and associated projects) had been able to adapt to local and national contexts and circumstances (see COVID-19 sub-theme below), and how key stakeholders (i.e. guide and crew) could react quickly to these circumstances to benefit the overall approach. For example, this meant being able to a) develop proposals with organisations quickly to secure new funds, b) present evaluative information to funding partners to re-assure them that progress was made, and c) change governance structures to better support the vision of WCM. This need for adaptation was recognised by many of the interviewees. In contrast, the Falls Prevention Initiative (Case 1) perhaps resembled a more traditional approach to service delivery; it had key performance

For peer review only - http://bmjopen.bmj.com/site/about/guidelines.xhtml

indicators, it was held accountable by the funding organisation to these indicators and deliverables, and the relationship between funders and the providers was considered somewhat transactional. Here, we saw a clash between traditional- and the desired- ways of working within WCM. We Can Move appeared, according to interviewees, to work more effectively when it was able to alter its course in response to the ever-changing, choppy nature of the river (i.e. circumstances and context).

> *It doesn't feel within the [organisation] to… there's not that trust, I don't believe, from the [organisation] for us to adapt the programme because we are seeing a need to change and adapt and I think there's not that trust of, okay, we understand that like there is in some other areas of We Can Move. I think with this [the Falls Prevention Initiative] you have to justify and evidence every change you make... – Participant 8, Case 1, Implementation team*

> *It's all about the relationships isn't it and you can't – the organisations you might think you are best placed to work with might not want to work with you or you might not have the relationship established. So yes you kind of have to build that don't you, in the short term it might mean doing different things or working with different partners, because as you say that's where the appetite is and the energy is. – Participant 8, Case 2, National physical activity organisation*

The **COVID-19 pandemic** both foregrounded and bolstered the ability of WCM to adapt to changing contexts. Whilst creating many challenges for the implementation of WCM, COVID-19 was also a catalyst in enabling or necessitating multi-sectoral, multi-organisational working. This theme was particularly present within the REM analyses. For example, in the Falls Prevention Initiative (Case 1), the educational materials were disseminated more broadly than initially anticipated – and through new organisations – due to the requirement for many older adults to isolate in the early waves of the pandemic. More broadly across WCM (Case 3), the impact of COVID-19 meant that many organisations had to de-prioritise their involvement in WCM to remain operational. This subsequently meant that WCM had to alter its priorities which it was able to do in part because of its adaptive governance structures (i.e. the ways in which other organisations become involved). As new money became available across the system from central government and other national funding agencies, Active Gloucestershire, due to their credibility as an organisation and centrality within the local networks, were able to bring stakeholders together from multiple organisations to capitalise on these funding streams to support the health – and wider needs - of local populations. In turn, this then led to new areas of work, new relationships, and wider impacts of WCM across the system.

> *I guess COVID and needing to work in a new way just made people look up a bit [i.e. look outside of their organisational silo] because there was a need. Previously you had a service and then suddenly you didn't, so there was a gap which you couldn't fill with anything traditional. Why would you look up when you've got a working service, essentially? But when that need came,*

For peer review only - http://bmjopen.bmj.com/site/about/guidelines.xhtml

people were like, 'Okay, so what can we do differently?' and then that's when those links – people were like, 'Okay, I'll ask if he's got any ideas' or… - Participant 6, Case 1, NHS commissioning

3.3 Barriers to implementation

Four main barriers to the implementation of WCM were identified. These include challenges related to evaluation, issues surrounding opposing mindsets within WCM, misaligned understanding and expectations of WCM, and the negative implications of COVID-19.

The **evaluation** of WCM, and associated projects (e.g. the Falls Prevention Initiative and Community-based Initiative), was problematic for several reasons. Across the three case studies, there were inconsistent expectations around the purpose and role of the evaluation across WCM. For example, in Case 2, several interviewees stated that it was difficult to measure and capture the unanticipated impacts of a community-driven initiative (that did not have a predetermined project plan). In Case 1, interviewees disagreed on whether it was important to focus on the "hard numbers" associated with the Falls Prevention Initiative to determine the success of the project. For some, these numbers (i.e. reduction in number of people being admitted to hospital due to a fall) were the most important "success" measure, but for others, especially those closest to the implementation, believed that it was not feasible to monitor this or attribute change to the intervention directly. These issues around attribution of effect were also noted amongst the wider WCM stakeholders (i.e. Case 3).

They [senior council leaders] were literally grilling the community builders and I don't think I was meant to stand up, I was just in the gallery [of the Town Hall] viewing the whole thing and I just said, 'Look, can I speak a minute?' I said, 'At the end of the day this isn't something that you can just see happening overnight, you have to wait.' It's taken however long for me to get to where I am now, 'cause I'm doing, I suppose you could say, unofficial community building, but as a volunteer, I suppose, but it takes time. If I expect everything to happen overnight, I'll just give up and won't bother and so I think back to me, this is something that you can't just assess overnight and you've got your answers, you have to wait to see the positives that come out of it. – Participant 4, Case 2, Community member

This is closely entwined with the next barrier, a **clash of mindsets** within WCM. In part, it appeared that the expectations surrounding the evaluation were linked to the mindset and ethos of the individual, organisation and/or sector. We Can Move was advocating for an alternative way of working across the system, meaning that multiple organisations are involved in the implementation and adaptation of WCM over time. However, each organisation coming aboard WCM had their own ways of working, and in particular, some organisations adopted transactional ways of working. This meant that these organisations were typically accustomed to commissioning services, setting key

For peer review only - http://bmjopen.bmj.com/site/about/guidelines.xhtml

performance indicators (thus linked to evaluation issues above), having regular performance update meetings, and therefore having a hierarchical relationship between commissioner and provider. By and large, this way of working was at odds with WCMs – referred to as "*the old and new ways of working*" (Participant 5, Case 3, NHS commissioning). This picture is nuanced though. In both the Falls Prevention Initiative (Case 1) and WCM (Case 3) interviews, several interviewees stated that whilst their own preferred way of working aligned with WCM, they found themselves working in organisations that were predominantly, and traditionally, transactional. This created significant challenges for these individuals – who were often the conduits and brokers between WCM and their organisation – as they held a mediating role, trying to re-iterate the purpose of WCM to their organisational leaders whilst simultaneously trying to demonstrate the value of WCM and reassure leaders that their investment in WCM was useful.

Misaligned understandings and expectations surrounding WCM were another common barrier mentioned in Cases 1 and 3. Several interviewees, especially those who were more peripheral to WCM, stated that they did not understand what WCM was and how it differed from Active Gloucestershire. We Can Move was seen as intangible by many of these stakeholders, and in part, this was due to what they perceived as complicated messaging. The premise of WCM was not consistently communicated, or understood, by those involved and this contributed to further misunderstanding and confusion. Similarly, if interviewees were predominantly involved in a project (e.g. Falls Prevention Initiative or Community-based Initiative) rather than WCM more broadly, they often struggled to see how these particular projects linked with the wider work of WCM. And so, when asked what their role was within WCM, these interviewees either a) did not see themselves having a role, or b) did not know what was expected of them. Whilst one of the strengths of Active Gloucestershire, noted by interviewees, was their communication with wider stakeholders, this was not consistent.

The last barrier was that of **COVID-19**. It is important to note that most of the data collection for the second case study, the Community-based Initiative, was undertaken before the pandemic began, and so there was very little data on this in the data set. In the other two cases, COVID-19 meant that much of the planned implementation work was paused or cancelled, and WCM and the Falls Prevention Initiative were required to adapt in response to the imposed restrictions (see strategies section). The primary reasons for this stagnation were that wider stakeholders reverted back to their silos because their organisational priorities changes (i.e. the need to "*survive*" – Participant 7, Case 3, Local third sector organisation) and many organisations had to furlough staff. This prevented progress being made between stakeholders who were involved in WCM before the pandemic began.

For peer review only

It's just been such a rubbish year and I think that that has overshadowed and become even more of an obstacle to getting stuff going because you've got the people that are shielding for themselves or others [from COVID-19], the low mood increase, lack of motivation, the disinterest in doing anything because things almost seem pointless… You've got to understand it and it's been impossible, I would say, to get things as good as they could be. – Participant 6, Case 3, Local third sector organisation

3.4 Contextual factors

Contextual factors are those which are not part of the intervention, or were already established pre-implementation, but affect its subsequent implementation and impact (35, 36). Interviewees often described the **organisational strengths of Active Gloucestershire**; the organisation was said to be well-connected, respected and a reliable source of knowledge within the county. In short, Active Gloucestershire were already well placed to guide the WCM raft, to be the backbone organisation. That is, the organisation that facilitate, and are accountable for, the implementation (and adaptation) of WCM.

"The result of one of those conversations [with senior leaders from the county] was the head of commissioning said, 'We need your people off furlough,' and that led to us working over the summer holidays with children that were in social care, have got PTSD, all these things… We would have found our way to that table, but it would have taken a lot of leg work, whereas Active Gloucestershire have got these inroads to these various different partners and have been great at keeping that door open for charities like us." - Participant 8, Case 3, Local third sector organisation

Stakeholders also suggested that WCM was **pushing on a slightly open door**. The council, and several national and local funders, were becoming more receptive to systems approaches. There was already a history of falls prevention work in the county (Case 1), a growing interest and application of place-based and asset-based community development (Case 2), and a rich and cohesive voluntary sector within the Community-based Initiative area (Case 2). Systems approaches have also been adopted in Gloucestershire to other issues such as obesity and ACEs. That said, some organisations involved in Case 1 were said to work in a transactional manner whereby Active Gloucestershire are accountable to a commissioner. Organisational engagement in WCM was influenced in part by their institutionalised ways of working. Thus, linking back to the analogy, Active Gloucestershire had prior knowledge of some of the river conditions before commencing WCM. They also had several crew members, or at least their respective organisations, warmed up and ready to go.

"It's almost like you want to put a heat map over the county, or a magnet, and there are some particular individuals that are really, really interested in systems change. They can see the

For peer review only - http://bmjopen.bmj.com/site/about/guidelines.xhtml

> *strategic benefit of it. However, many of those individuals are stuck in traditional institutions and therefore really struggle to innovate, so they will try and be supportive but there'll be certain hoops that they have to jump through or political issues that they're encountering" – Participant 3, Case 3, Local third sector organisation*

3.5 Impacts and outcomes

We derived four main types of impact from the analysis: 1) physical activity-related impacts, 2) relational impacts, 3) sectoral impacts, and 4) null or negative impacts. It is important to note here that the **physical activity-related impacts** were not objectively measured, but rather they were perceived changes in physical activity noted by the interviewees or REM participants. Interviewees – especially in relation to Case 2 – suggested that the opportunities for physical activity amongst Black, Asian and Minority Ethnic (BAME) women had improved. This was also reported in relation to the older adults (Case 1) who received the educational materials from the Falls Prevention Initiative. More broadly though, in Case 3 and through the REM analysis, we identified many instances where physical activity, for individuals, communities, and populations, may have been influenced.

> *They had over 100 women get involved cross all the different activities, and mostly BAME women who wouldn't engage, or who aren't very well connected. So, when the different women come in, they have carried on as well. Over 100 women. That's not bad when you started off with just 12 women who wouldn't engage with other communities. – Participant 1, Case 2, City council*

The main impacts however, as per this analysis, were **relational** and **sectoral**. There was said to be improved cohesion and collaboration between stakeholders involved in, or on the periphery of, WCM, and many examples were given. As previously noted, we found that at least 82 organisations were involved in WCM and that 23 networks were attended or organised. Being part of WCM enabled stakeholders to feel like they were part of something bigger, an initiative or way of working that went beyond their own organisation. This was supported by the generation and dissemination of new funding via WCM – enabling organisations to come together. It was through these new or improved relationships that we saw cross-sectoral impacts; for example, the work of WCM aligned with and supported the work being undertaken on mental health, air quality, tackling inequality, and town planning (among others). And many commented that their own mindset (i.e. a way of thinking) and that of their senior leaders had started to change due, in part, to their involvement in WCM. Lastly, through the REM, we found that WCM and physical activity had been integrated into a minimum of six policies across the county, ranging from the Health and Wellbeing Strategy (county wide) to City Planning policies (City wide) to Transport Planning policies (City wide).

For peer review only - http://bmjopen.bmj.com/site/about/guidelines.xhtml

Like I say, there's other people that you see and other ladies and it's finding out what they're doing as well. It's doing things together as a group. They're not only like – it's not to be said, we're going to [steering group member A] and nobody knew about [steering group member B]. It's given [steering group member C] the opportunity to get to know the people in the community in this area. – Participant 9, Case 2, Community member

There were some **negative or null impacts** reported too. Some interviewees stated that there had not been any beneficial impacts on them or their organisation, and that this was due to a lack of engagement or an inability to engage due to COVID-19. This meant that some of the organisations which were involved in WCM were still working, largely, in silos. Other null impacts were more specific to individual case studies, for example, some interviewees said that the Falls Prevention Initiative had not improved the number of older adults being referred to strength and balance classes.

I thought after the [systems] mapping exercise it was so nice to bring all those organisations together, really beneficial. We had some really positive conversations and I was really hoping there would be some follow-up to that so that I can then start to work with more organisations and look at how we fit as part of this wider approach and not just in silo. I think, for me, that's what We Can Move is supposed to be. It's to bring everyone together and, yeah, create a system change, whereas we're still just cracking on as we're doing. – Participant 7, Case 3, Local third sector organisation

4. Discussion

There is currently limited evidence available regarding the implementation of systems approaches in the field of physical activity and across public health more broadly (27). This paper highlights the key strategies and contextual factors that were associated with the implementation of WCM in Gloucestershire. It also illuminates the types of impact experienced within the first three years of implementation, but notes that there were several barriers which influenced the implementation, and thus, the impact of WCM. Given that there is a paucity of research, this paper provides an important, novel, and timely contribution to the evidence base, with several implications for future practice and evaluation.

4.1 What's needed to start and maintain a systems approach?

Our study identified several important factors to consider when initiating and maintain a systems approach. Understanding, and talking to, the motivations of the wider stakeholders across the system was imperative to kick-starting this work. Active Gloucestershire already had strong relationships across the county, but nevertheless, they had to draw people into WCM. This meant understanding

For peer review only - http://bmjopen.bmj.com/site/about/guidelines.xhtml

the strategic priorities of other stakeholders and organisations, and in doing so, talking to the shared benefits that could be realised through their involvement in WCM. For example, many organisations were looking to do more partnership work across the county. Active Gloucestershire were able to highlight that a core benefit from engagement in WCM would be that organisations are encouraged to work more closely together, to achieve greater impact, and improve the use of scarce resources.

The initial and successful engagement of stakeholders helped WCM to develop the "right" crew (linked to the rafting analogy). Understanding the initial motivations of the wider stakeholders, and speaking to the shared benefits, brought them aboard. However, once aboard, the role of Active Gloucestershire (as the guide of the raft) was to help stakeholders see the bigger picture – to recognise that their own work and priorities are intertwined with others – and to consider the ways in which they could work together. Formal networking events and meetings, as well as informal relationship brokering, were methods used by Active Gloucestershire to encourage collaboration and a shared vision between stakeholders. These are not new phenomena and are deemed by many to be core ingredients of a systems approach and primary outcomes of systems or adaptive leadership (26, 27, 34, 44-48). In effect, Active Gloucestershire were developing the crew and encouraging them to paddle together towards a common goal.

Adaptation is one of the main characteristics of a complex adaptive system (49), and therefore has to be accounted for and anticipated when implementing a systems approach. The impact of COVID-19 caused substantial and rapid adaptations in the local, national, and international systems. For WCM, it meant that many pre-planned initiatives had to be paused (e.g. engagement with schools), but it also brought about new opportunities for collaborative working which may otherwise not have occurred. Given the flexibility in the design of WCM, it was able to adapt accordingly to these turbulences rather than coming to a halt. It did mean though that some stakeholders, such as the local public health team, became less involved (as they were integral to the COVID-19 response) whilst other organisations, such as local faith groups, became more involved (e.g. through distribution of food and activity packs to the community during COVID-19 lockdown restrictions). We referred to this adaptability of WCM as "reading the river". Donella Meadows refers to this as "dancing with systems" (50).

4.2 What type of impacts may we expect from a systems approach?

Capturing the diversity of impacts arising from a systems approach is difficult (51). Within this evaluation, we brought together two methods (REM and semi-structured interviews) which we found helpful in trying to better understand the anticipated and unanticipated impacts of WCM across the Gloucestershire systems – i.e. the adaptive and emergent nature of the system and of WCM. Ripple

For peer review only - http://bmjopen.bmj.com/site/about/guidelines.xhtml

Effects Mapping allowed us qualitatively to capture, close to real time, many of the activities and impacts associated with WCM over a two-year period. The interviews were then used to gather further information about these impacts, and to learn more about how these impacts may have occurred. Our analysis showed that there were perceived changes in physical activity among particular communities (e.g. Muslim women within an area of Gloucester – Case 2), and changes to the physical activity environment (e.g. changing policies around access to leisure facilities and changes to physical activity opportunities in school). However, as others have found (52), system approaches such as WCM foster new relationships – and cohesion between stakeholders – across the system.

Using the REM, we were also able to demonstrate where WCM benefited other strategic priorities and made intangible impacts, tangible. We noted that WCM was embedded into six policies and had aligned with 22 other agendas (including air quality, town planning, inequalities, and mental health). In the 2019 Lancet commission (23), Swinburn *et al*. refer to such initiatives as double or triple duty actions – those which contribute towards several outcomes at once. Linking back to the initial role of Active Gloucestershire, and perhaps other backbone organisations, we were able to see how the appeal of shared benefits as a way of initially engaging stakeholders can be realised through a systems approach. We also documented less tangible, or softer, impacts through REM which included the changing mindsets of senior leaders within the system, enhanced and more cohesive relationships between partners, and the WCM Theory of Change being considered in other areas of the UK. While the relational and mindset aspects of these intangible outcomes are aspired when implementing a systems approach (22, 26, 44, 51), they are seldom reported on, but here, REM enabled us to surface, capture and analyse these as part of the WCM evaluation.

4.3 What might moderate the implementation and impact of a systems approach?

We highlighted several key factors which influenced the implementation and impact of WCM, and these would likely occur in other areas who are aiming to adopt a systems approach. In line with characteristics of a complex adaptive system (53), it is important for stakeholders to assess the "initial conditions" in which the systems approach is to be implemented (54, 55). Gloucestershire – and many of the organisations within it – was a county which was somewhat primed for WCM; some people (e.g. council staff, NHS staff, Voluntary and Community Sector organisations) were familiar with systems approaches, the associated jargon, and many had already established cross-sectoral relationships. Similarly, Active Gloucestershire were a well-respected and credible organisation. Linking back to the rafting analogy, WCM started out with a sturdy raft, a strong guide, and a forming crew. Some wishing to adopt a systems approach may also have strong foundations to build on, whilst others may not, and

For peer review only - http://bmjopen.bmj.com/site/about/guidelines.xhtml

so prospective implementers and evaluators need to be mindful of the starting point and anticipate what impact this may have on early efforts (35, 56, 57).

Once WCM commenced, other challenges arose which influenced the rate at which it built momentum. A major challenge was a clash of mindsets, between those wishing to implement – or be involved in – a systems approach and those wanting to retain a more traditional service commissioning / delivery hierarchy. This becomes more problematic if these are institutional or sectoral mindsets (e.g. across healthcare) rather than that of a particular individual (e.g. a manager within healthcare). Historically in the UK, and elsewhere, public and population health (which include physical activity) has been delivered using a traditional medical model (18, 58-60). In turn, this means that many issues – such as low levels of physical activity – are treated and prevented akin to other medical issues. Thus, this often leads to the funding, commissioning and provision of individual focused services and programmes – for example, physical activity sessions, health promotion campaigns, weight management programmes etc. These medicalised approaches have been deeply engrained within healthcare and public health, and in part, have contributed to siloed working (61, 62). In this evaluation, despite endorsement of WCM, some stakeholders and organisations appeared uncomfortable with the WCM approach; it meant devolved power, shared responsibility and ownership, less tangible outcomes, and accepting uncertainty. These are not findings unique to this context. Crane *et al.* (62) highlighted this as one of four major challenges facing the sustainment of population health programmes, and Alderwick *et al.* (48) noted it as a significant hurdle facing the collaboration between health care and non-health care organisations. Many see bold, and systems, leadership as a means of working through these challenges (44, 45, 47, 59, 62).

4.4 Strengths and Limitations

This study draws on the data from semi-structured interviews and REM. The latter represents a novel participatory approach to capture the wider impacts of a programme over time. We adapted the method, and report on the findings here, for the first time in the field of systems change in public health. Our robust methodological approach meant that we could gain in-depth insight into the complexity of WCM; through the methods used, the lines of inquiry, the analytical approach, and importantly, the use of an embedded researcher. It is due to these strengths that we believe the findings of this work will be highly relevant and useful to others.

This work is not without its limitations. The impact of the COVID-19 pandemic meant that most data collection was completed online. This likely affected the quality of the REM sessions as it reduced participation and interaction between stakeholders. It also meant that we reduced the length of the sessions, and so it is possible that in-person, longer REM sessions would have provided greater depth

For peer review only

For peer review only - http://bmjopen.bmj.com/site/about/guidelines.xhtml

to the outputs. Similarly, we only included the implementation team in the REM workshops to help bound our data collection. This limits the ripples within the outputs to those known to the implementation team. In retrospect, and if in person sessions were feasible, we would have included wider stakeholders in REM sessions to examine the impacts towards the periphery of WCM. Finally, this paper draws on two of the seven methods used to evaluate WCM. Whilst the semi-structured interviews and REM findings provide unique, and detailed, insights into WCM, the other methods (not reported on here) allowed us to understand stakeholder engagement (e.g. via stakeholder survey), the complexity of physical inactivity (e.g. via participatory systems mapping), and participation in events (e.g. via secondary analysis of routine data). It was not feasible to include data from all of these sources in this paper, and those interested in the wider 25evaluation are encouraged to read Nobles *et al.,* (37)

4.5 Future considerations

This research has highlighted several important implications and considerations for those implementing or evaluating systems approaches in Public Health. First is the importance of understanding context. Both implementers and evaluators should spend considerable time understanding the initial conditions within the local systems (e.g. organisations, relationships, key actors, local action and policy, historic socio-political factors etc…). These conditions – as shown in the results – contributed to the initial implementation of WCM. Second is the need for stakeholders to determine what lies within the scope of the evaluation – acknowledging that different people will value different elements of the systems approach. Where possible, stakeholders should seek to establish the evaluation boundaries and foci early and clearly communicate these to all involved stakeholders. Note too that these boundaries and foci may change over time. Building on this, third is that evaluators may wish to examine the different perspectives of wider stakeholders involved in the approach. Aim to understand their motivations for engagement, what they know of the approach, how they anticipate it to work, and what impacts and outcomes would be of value to them. This may help ensure that the evaluation is meaningful to a range of actors, not just those who are interested in the primary outcome (i.e. increasing population physical activity). A fourth consideration is to question how principles of systems science can be further embedded in the implementation and evaluation of these approaches. For example, how can evaluations gather more information on the adaptive nature of the system, and of systems change efforts? How can implementers ensure – or enhance the likelihood of – identifying and changing deep-rooted leverage points within the system? How can both parties ensure that the evaluation contributes to the continued learning and refinement

For peer review only - http://bmjopen.bmj.com/site/about/guidelines.xhtml

of the systems approach? And fifth, as others point out (57, 63), further methodological innovation and publication of similar evaluations are warranted to advance the knowledge base around Public Health-orientated systems approaches.

5. Conclusions

This paper is one of few to report on the evaluation of a physical activity-orientated systems approach. We highlighted several important strategies and contextual factors which enabled WCM to function as it did, and moreover, captured a wide range of impacts, both anticipated and unanticipated, that were associated with its implementation. These findings are important for the field. They present an honest and robust account of a wide range of stakeholder perspectives who were engaged in, or affected by, a systems approach. They highlight some of the barriers experienced when implementing the approach, and it is likely that others will come across these too in the future. This paper advances the evidence base and provides the findings from a novel, participatory method of REM which was triangulated with interview findings. Further research is needed to continue building the evidence base in this area.

For peer review only

For peer review only - http://bmjopen.bmj.com/site/about/guidelines.xhtml

Additional information

Funding statement: The evaluation of We Can Move was commissioned by Active Gloucestershire. Some of James Nobles', Russell Jago's, and Sabi Redwood's time was supported by the National Institute for Health Research (NIHR) Applied Research Collaboration West (NIHR ARC West). The views expressed in this article are those of the author(s) and not necessarily those of the NIHR or the Department of Health and Social Care. The Article Processing Charge was funded through NIHR ARC West. Award / grant number: N/A

Competing interests: The authors have no competing interests to declare.

Author contributions: JN, SR, RJ & CFos conceptualised the paper, and JN wrote the first draft of the manuscript. All authors (JN, CFox, AIW, TB, SR, RJ & CFos) provided substantial input into later drafts of the manuscript. All authors refined and discussed subsequent versions of the manuscript before approving the final version.

Data sharing statement: No additional data available.

Ethics approval: Ethics approval was granted by the Faculty of Health Sciences, University of Bristol (*Ref*. 91145) and all participants provided written informed consent.

For peer review only

References

1. Guthold R, Stevens GA, Riley LM, Bull FC. Global trends in insufficient physical activity among adolescents: a pooled analysis of 298 population-based surveys with 1·6 million participants. The Lancet Child & Adolescent Health. 2020;4(1):23-35.
2. Guthold R, Stevens GA, Riley LM, Bull FC. Worldwide trends in insufficient physical activity from 2001 to 2016: a pooled analysis of 358 population-based surveys with 1·9 million participants. The Lancet Global Health. 2018;6(10):e1077-e86.
3. Sport England. Active Lives Adult Survey - May 2019/20 Report. London, UK; 2020.
4. U.S. Department of Health and Human Services. 2018 Physical activity guidelines advisory committee scientific report. 2018.
5. Lee IM, Shiroma EJ, Lobelo F, Puska P, Blair SN, Katzmarzyk PT. Effect of physical inactivity on major non-communicable diseases worldwide: an analysis of burden of disease and life expectancy. The Lancet. 2012;380(9838):219-29.
6. International Society for Physical Activity and Health (ISPAH). ISPAH's Eight Investments That Work for Physical Activity. 2020.
7. Ding D, Lawson KD, Kolbe-Alexander TL, Finkelstein EA, Katzmarzyk PT, van Mechelen W, et al. The economic burden of physical inactivity: a global analysis of major non-communicable diseases. The Lancet. 2016;388(10051):1311-24.
8. Bull FC, Al-Ansari SS, Biddle S, Borodulin K, Buman MP, Cardon G, et al. World Health Organization 2020 guidelines on physical activity and sedentary behaviour. British Journal of Sports Medicine. 2020;54(24):1451-62.
9. Jáuregui A, Lambert EV, Panter J, Moore C, Salvo D. Scaling up urban infrastructure for physical activity in the COVID-19 pandemic and beyond. The Lancet. 2021;398(10298):370-2.
10. Shilton T, McLaughlin M, Reece L, Chalkley A, Gomersall S, Schipperijn J, et al. ISPAH's Eight Investments That Work for Physical Activity. In: Health ISfPAa, editor. 2020.
11. Rutter H, Cavill N, Bauman AB, F. Systems approaches to global and national physical activity plans. Bulletin of the World Health Organisation. 2019;97:162-5.
12. Cavill N, Richardson D, Faghy M, Bussell C, Rutter H. Using system mapping to help plan and implement city-wide action to promote physical activity. J Public Health Res. 2020;9(3):1759-.
13. World Health Organisation. Global Action Plan for Physical Activity 2018-2020. Geneva, Switzerland; 2018.
14. Tibbitts B, Willis K, Reid T, Sebire SJ, Campbell R, Kipping RR, et al. Considerations for Individual-Level Versus Whole-School Physical Activity Interventions: Stakeholder Perspectives. International Journal of Environmental Research and Public Health. 2021;18(14):7628.
15. Adams J, Mytton O, White M, Monsivais P. Why Are Some Population Interventions for Diet and Obesity More Equitable and Effective Than Others? The Role of Individual Agency. PLOS Medicine. 2016;13(4):e1001990.
16. Atkins L, Kelly MP, Littleford C, Leng G, Michie S. Reversing the pipeline? Implementing public health evidence-based guidance in english local government. Implementation Science. 2017;12(1):63.
17. Swinburn BA, Sacks G, Hall KD, McPherson K, Finegood DT, Moodie ML, et al. The global obesity pandemic: shaped by global drivers and local environments. The Lancet. 2011;378(9793):804-14.
18. Rutter H, Savona N, Glonti K, Bibby J, Cummins S, Finegood DT, et al. The need for a complex systems model of evidence for public health. The Lancet. 2017;390(10112):2602-4.
19. Parkhurst JO, Abeysinghe S. What Constitutes "Good" Evidence for Public Health and Social Policy-making? From Hierarchies to Appropriateness. Social Epistemology. 2016;30(5-6):665-79.
20. McMahon NE. What shapes local health system actors' thinking and action on social inequalities in health? A meta-ethnography. Social Theory & Health. 2022.

For peer review only - http://bmjopen.bmj.com/site/about/guidelines.xhtml

21. Lorenc T, Petticrew M, Welch V, Tugwell P. What types of interventions generate inequalities? Evidence from systematic reviews. Journal of Epidemiology and Community Health. 2013;67(2):190-3.
22. Nobles JD, Radley D, Mytton OT. The Action Scales Model: A conceptual tool to identify key points for action within complex adaptive systems. Perspectives in Public Health. 2021:17579139211006747.
23. Swinburn BA, Kraak VI, Allender S, Atkins VJ, Baker PI, Bogard JR, et al. The Global Syndemic of Obesity, Undernutrition, and Climate Change: The Lancet Commission report. The Lancet. 2019;393(10173):791-846.
24. Michie S, van Stralen MM, West R. The behaviour change wheel: A new method for characterising and designing behaviour change interventions. Implementation Science. 2011;6(1):42.
25. Fortescue-Webb D, Evans T, Hanss K, Lamont T. Moving Matters - Interventions To Increase Physical Activity. 2019.
26. Public Health England. Whole systems approach to obesity programme: A guide to support local approaches to promoting healthy weight. London, UK.: PHE Publications; 2019.
27. Bagnall A-M, Radley D, Jones R, Gately P, Nobles J, Van Dijk M, et al. Whole systems approaches to obesity and other complex public health challenges: a systematic review. BMC Public Health. 2019;19(1):8.
28. Rutter H, Glonti K. Towards a new model of evidence for public health. The Lancet. 2016;388:S7.
29. Sport England. People and places; the story of doing it differently. London, UK; 2021.
30. Karacabeyli D, Allender S, Pinkney S, Amed S. Evaluation of complex community-based childhood obesity prevention interventions. Obesity Reviews. 2018;19(8):1080-92.
31. Public Health England. Whole systems approach to obesity programme: Learning from co-producing and testing the guide and resources. London, UK.: PHE Publications; 2019.
32. Finegood DT. Can We Build an Evidence Base on the Impact of Systems Thinking for Wicked Problems?; Comment on "What Can Policy-Makers Get Out of Systems Thinking? Policy Partners' Experiences of a Systems-Focused Research Collaboration in Preventive Health". International Journal of Health Policy and Management. 2020:-.
33. Bellew W, Smith BJ, Nau T, Lee K, Reece L, Bauman A. Whole of Systems Approaches to Physical Activity Policy and Practice in Australia: The ASAPa Project Overview and Initial Systems Map. Journal of Physical Activity and Health. 2020;17(1):68-73.
34. Kania J, Kramer M. Collective Impact. Stanford Social Innovation Review. 2011.
35. Skivington K, Matthews L, Simpson SA, Craig P, Baird J, Blazeby JM, et al. A new framework for developing and evaluating complex interventions: update of Medical Research Council guidance. BMJ. 2021;374:n2061.
36. Moore GF, Audrey S, Barker M, Bond L, Bonell C, Hardeman W, et al. Process evaluation of complex interventions: Medical Research Council guidance. BMJ : British Medical Journal. 2015;350:h1258.
37. Nobles J, Redwood S, Jago R, Hill L, Mamluk L, Foster C. Evaluation of we can move: April 2019 – April 2021. 2021.
38. Nobles J, Wheeler J, Dunleavy-Harris K, Holmes R, Inman-Ward A, Potts A, et al. Ripple effects mapping: capturing the wider impacts of systems change efforts in public health. BMC Med Res Methodol. 2022;22(1):72.
39. Potts AJ, Nobles J, Shearn K, Danks K, Frith G. Embedded Researchers as Part of a Whole Systems Approach to Physical Activity: Reflections and Recommendations. Systems. 2022;10(3):69.
40. Chazdon S, Emery M, Hansen D, Higgins L, Sero R. A Field Guide to Ripple Effects Mapping. Minneapolis, USA: University of Minnesota Libraries Publishing; 2017.
41. Cooperrider D, Srivastva S. Appreciative inquiry and organisational life. Research in Organisational Change and Development. 1: JAI Press; 1987. p. 129-69.

For peer review only

42. Gale NK, Heath G, Cameron E, Rashid S, Redwood S. Using the framework method for the analysis of qualitative data in multi-disciplinary health research. BMC Med Res Methodol. 2013;13(1):117.
43. Sparkes AC, Smith B. Qualitative Research Methods in Sport, Exercise and Health. London, UK: Routledge; 2013.
44. Baugh Littlejohns L, Wilson A. Strengthening complex systems for chronic disease prevention: a systematic review. BMC Public Health. 2019;19(1):729.
45. Stansfield J, South J, Mapplethorpe T. What are the elements of a whole system approach to community-centred public health? A qualitative study with public health leaders in England's local authority areas. BMJ Open. 2020;10(8):e036044.
46. Bird EL, Evans D, Gray S, Atherton E, Blackshaw J, Brannan M, et al. Opportunities to engage health system leaders in whole systems approaches to physical activity in England. BMC Public Health. 2022;22(1):254.
47. Bigland C, Evans D, Bolden R, Rae M. Systems leadership in practice: thematic insights from three public health case studies. BMC Public Health. 2020;20(1):1735.
48. Alderwick H, Hutchings A, Briggs A, Mays N. The impacts of collaboration between local health care and non-health care organizations and factors shaping how they work: a systematic review of reviews. BMC Public Health. 2021;21(1):753.
49. Finegood DT. The Complex Systems Science of Obesity. In: Cawley J, editor. The Oxford Handbook of the Social Science of Obesity. New York, USA. : Oxford University Press, Inc.; 2011.
50. Meadows D, Wright D. Thinking in Systems: A Primer: Chelsea Green Publishing; 2008.
51. Jebb SF, D, Diez Roux A, Rutter H, Clarkson J, Frank J, Roos N, et al. Systems-based approaches in public health: Where next? UK Academy of Medical Sciences & Canadian Academy of Health Sciences.,; 2021.
52. Gadsby EW, Hotham S, Eida T, Lawrence C, Merritt R. Impact of a community-based pilot intervention to tackle childhood obesity: a 'whole-system approach' case study. BMC Public Health. 2020;20(1):1818.
53. FSG. Evaluating Complexity: Propositions for Improving Practice. 2017.
54. Walton M. Applying complexity theory: A review to inform evaluation design. Evaluation and Program Planning. 2014;45:119-26.
55. Hawe P, Bond L, Butler H. Knowledge theories can inform evaluation practice: What can a complexity lens add? New Directions for Evaluation. 2009;2009(124):89-100.
56. Matheson A, Walton M, Gray R, Lindberg K, Shanthakumar M, Fyfe C, et al. Evaluating a community-based public health intervention using a complex systems approach. Journal of Public Health. 2017;40(3):606-13.
57. McGill E, Marks D, Er V, Penney T, Petticrew M, Egan M. Qualitative process evaluation from a complex systems perspective: A systematic review and framework for public health evaluators. PLOS Medicine. 2020;17(11):e1003368.
58. Orton LC, Lloyd-Williams F, Taylor-Robinson DC, Moonan M, O'Flaherty M, Capewell S. Prioritising public health: a qualitative study of decision making to reduce health inequalities. BMC Public Health. 2011;11(1):821.
59. Hunter D, Perkins N. Partnership Working in Public Health: The Implications for Governance of a Systems Approach. Journal of Health Services Research & Policy. 2012;17(2_suppl):45-52.
60. Blackman T, Harrington B, Elliott E, Greene A, Hunter DJ, Marks L, et al. Framing health inequalities for local intervention: comparative case studies. Sociology of Health & Illness. 2012;34(1):49-63.
61. Lynch J. Reframing inequality? The health inequalities turn as a dangerous frame shift. Journal of Public Health. 2017;39(4):653-60.
62. Crane M, Nathan N, McKay H, Lee K, Wiggers J, Bauman A. The Sustainment of Population Health Programs Requires a Systems Approach. Health Research Policy and Systems. 2022.

For peer review only

63. McGill E, Er V, Penney T, Egan M, White M, Meier P, et al. Evaluation of public health interventions from a complex systems perspective: A research methods review. Social Science & Medicine. 2021;272:113697.

For peer review only

For peer review only - http://bmjopen.bmj.com/site/about/guidelines.xhtml

Figure legends

Figure 1: Simple REM Example

For peer review only

For peer review only - http://bmjopen.bmj.com/site/about/guidelines.xhtml

Figure 1

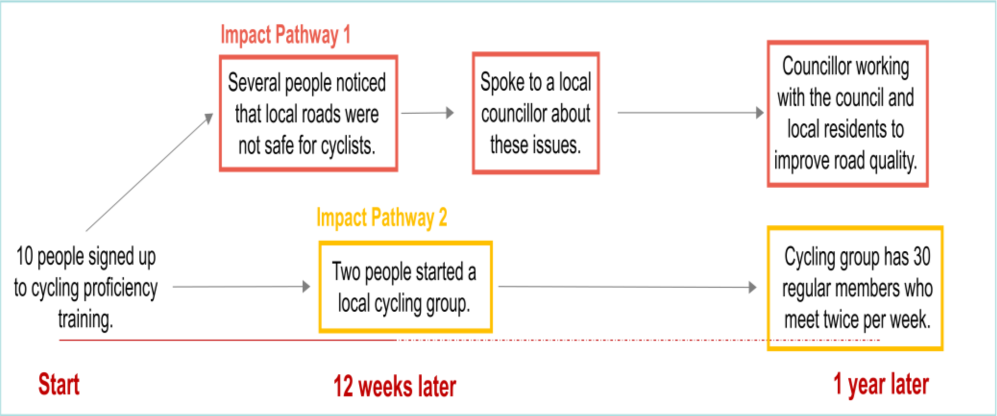
Impact Pathway 1
Several people noticed that local roads were not safe for cyclists.
Spoke to a local councillor about these issues.
Councillor working with the council and local residents to improve road quality.
Impact Pathway 2
10 people signed up to cycling proficiency training.
Two people started a local cycling group.
Cycling group has 30 regular members who meet twice per week.
Start
12 weeks later
1 year later

Supplement I - COREQ Checklist: Nobles et al. Implementing a Whole Systems Approach to Increase Physical Activity

Domain 1: Research team and reflexivity

Personal Characteristics

1. *Interviewer/facilitator*

James Nobles

2. *Credentials*

Ph.D.

3. *Occupation*

Research Fellow in Behavioural and Qualitative Science at the University of Bristol.

4. *Gender*

Male.

5. *Experience and training*

Seven years of qualitative research experience.

Relationship with participants

6. *Relationship established*

A relationship was present with some of the people who were interviewed / worked with for this project. James Nobles was an embedded researcher at Active Gloucestershire (the organisation who facilitate WCM), and so spent one day per week initially in their offices. During this time, he attended many meetings, workshops and events, at which he met some of the people who were interviewed. In all instances, the (prospective) interviewee knew that James' role was that of an evaluator and that he did not work for Active Gloucestershire. A concise explanation of this is offered on page 5, section 2.1.

7. *Participant knowledge of the Interviewer*

Where relationships were already established prior to the interview taking place, the researcher had always made clear that they were evaluating the implementation of WCM. James spoke about the different aspects of the evaluation (e.g. the research questions, the methods, the outputs etc..) at these meetings. It was important that stakeholders, some of which were future interviewees, understood why James was present in the meetings, workshops or events. A concise explanation of this is offered on page 5, section 2.1.

8. *Interviewer characteristics*

James had a pre-existing interest in the design, delivery and evaluation of systems approaches in Public Health. He had previously been involved in the creation of a toolkit that Local Government Authorities in England could use to design their own whole systems approach to prevent and

manage overweight and obesity. The topic of physical activity was somewhat new prior to starting the evaluation of WCM in April 2019, however other members of the team are international experts in physical activity research. Moreover, he – and the wider project team – were aware that the previous approaches to improving physical activity had largely been ineffective in bringing about long-term, sustainable behaviour change. It is worth stating here though that the purpose of the WCM evaluation was not to determine if the population levels of physical activity changed as a result of its delivery, but rather it was mainly concerned with the learning that arises from the implementation of WCM.

Domain 2: Study design

Theoretical framework

9. *Methodological orientation and Theory* - What methodological orientation was stated to underpin the study? e.g. grounded theory, discourse analysis, ethnography, phenomenology, content analysis

This is a multi-method qualitative evaluation study analysed using a framework and content approach. A concise explanation of this is offered on page 5, section 2.1.

Participant selection

10. *Sampling*

All interview participants were purposefully sampled, via one of three means:

- First, the researcher worked with members of the Active Gloucestershire team to identify people who had been involved in, or affected by, the WCM programme.
- Second, the researcher analysed the Ripple Effects Mapping outputs to identify other people or organisations who may have been involved in the periphery of WCM. Ripple Effects Mapping has been described in the main paper.
- Third, the researcher conducted a secondary analysis of a stakeholder survey that was disseminated by Active Gloucestershire amongst their contacts. The researcher looked at the responses to open-ended questions. These respondents were then sent an invitation to partake in an interview by a member of the Active Gloucestershire team.

Ripple Effects Mapping participants comprised of implementation staff (i.e. those involved in the design and delivery of We Can Move). A small group of community residents also took part in an REM workshop, as described in the paper, and all were involved in the implementation of the community-based initiative.

A concise explanation of this is offered on page 7-8, section 2.3.

11. *Method of approach*

All participants were contacted via email in the first instance. They were provided with an overview of the evaluation, a description around the purpose of the interview, and then further information was included in a participant information sheet. Those who were then interested in the study were sent a consent form to complete. If participants had further questions, they were able to contact the researcher directly via phone or video-call software.

The ethical approval statement is provided on page 26.

12. *Sample size*

Interview data from thirty- one people were included in this study.

The justification for this sample size is provided on page 7, section 2.3.

13. *Non-participation* - How many people refused to participate or dropped out? Reasons?

No one refused to take part in an interview or REM session as far as we recall.

Setting

14. *Setting of data collection*

All interview data was collected either over the phone (i.e. telephone interviews) or via video-call software (e.g. Microsoft Teams or Zoom).

Initial REM sessions were completed via in-person workshops, and follow up workshops were all completed online using Microsoft Teams or Zoom.

A concise explanation of this is offered on page 7, section 2.3.

15. *Presence of non-participants* - Was anyone else present besides the participants and researchers?

No

16. *Description of sample* - What are the important characteristics of the sample? e.g. demographic data, date

The reporting of this is offered on page 10, section 3.

Data collection

17. *Interview guide* - Were questions, prompts, guides provided by the authors? Was it pilot tested?

The interview guide is provided as a supplement to the manuscript. The interview guide was developed by the co-authors of the paper who have extensive experience in qualitative and evaluation research. The questions were designed so as to specifically probe into areas of interest. The REM outputs, as described in the paper, were also used to probe for further information in the interviews.

A concise statement of this is provided on page 7, section 2.3.

18. *Repeat interviews* - Were repeat interviews carried out? If yes, how many?

No

19. *Audio/visual recording* - Did the research use audio or visual recording to collect the data?

Yes – interviews were audio recorded, and REM workshops (online only) were visually and audio recorded.

Concise statements of this are provided on page 7-8, section 2.3.

20. *Field notes* - Were field notes made during and/or after the interview or focus group?

Yes. These were predominantly made to facilitate or bolster the lines of inquiry when carrying out the interview (e.g. notes made on topics discussed so that aspects of the conversation could be revisited in the interview if necessary). Notes were not used to aid the analysis.

21. *Duration* - What was the duration of the interviews or focus

group? A concise statement of this is provided on page 7, section 2.3.

22. *Data saturation* - Was data saturation discussed?

No, we planned to complete a pragmatic number of interviews per case study (n=10).

23. *Transcripts returned* - Were transcripts returned to participants for comment and/or correction?

No, although upon receiving the transcripts, authors read these concurrent to the audio recording to ensure their accuracy.

Domain 3: analysis and findings

Data analysis

24. *Number of data coders* - How many data coders coded the data?

One primary coder and a second (CF) completed a separate analysis of the data from one case study. The two authors discussed their findings and came to an agreement on the most appropriate way to present these findings.

A concise statement of this is provided on page 9, section 2.4.

25. *Description of the coding tree* - Did authors provide a description of the coding tree?

No

26. *Derivation of themes* - Were themes identified in advance or derived from the data?

Yes – some themes were created based upon the main areas of exploration (e.g. mechanisms of change, contextual factors, impacts etc…), and others were derived from the data where required.

A concise explanation of this is provided on page 9, section 2.4.

27. *Software* - What software, if applicable, was used to manage the data?

~~NVIVO12~~NVivo12

28. *Participant checking* - Did participants provide feedback on the findings?

We reported the findings of this work back to the implementation team and wider stakeholders to ensure that it resonated with what they had experienced. Further details are available in the manuscript.

A concise statement of this is provided on page 9, section 2.4.

Reporting

29. *Quotations presented* - Were participant quotations presented to illustrate the themes / findings? Was each quotation identified? e.g. participant number

30. *Data and findings consistent* - Was there consistency between the data presented and the findings?

31. *Clarity of major themes* - Were major themes clearly presented in the findings?

N/A – this is based upon reviewer comments.

32. *Clarity of minor themes* - Is there a description of diverse cases or discussion of minor themes N/A – this is based upon reviewer comments.

Supplement II: Interview Topic Guide

1. Can you tell me about your **role** or involvement in "we can move"?

- How did your involvement commence?
- What were your reasons for getting involved?
- If not knowledgeable of "we can move", the role with Active Gloucestershire

2. What do you **perceive "we can move" to be**?

- What do you think of when you hear "we can move"?
- What do you know about it?

3. What were your **expectations** from "we can move"?

- How did you expect it to unfold?

4. How have you seen "we can move" **evolve over time**?

- How has this happened?
- What has contributed to these adaptations to occur?

5. What have been the **main successes** of "we can move" from your perspective?

- Why did you highlight these examples?

6. To what extent do you feel that "we can move" is **focused on the right things**?

7. [Facilitators] What has **helped** "we can move" to work as it does?

- i.e. what has helped to make it successful?
- What are the important ingredients?

[Barriers] What are the **challenges**, or have been the challenges, facing "we can move"?

- What has made it more difficult?

Contextual factors that may influence the projects?

8. What **impacts have you experienced** or heard about regarding "we can move"?

- i.e. impacts on self, society, organisation, policy etc….
- Expected or unexpected?
- How did the impact occur?
- Are these as you expected?
- Did they lead to any additional impacts?

9. For you, how could "we can move" **improve** or develop in the **future**?

- What would be required to help achieve this?

10. What do you **perceive your role to be** in "we can move" in the future?

For peer review only
